# Interfacial and Molecular Mechanisms of Pancreatic Lipase Modulation by Saponin-Rich Extracts with Antioxidant Activity

**DOI:** 10.3390/molecules31152600

**Published:** 2026-07-25

**Authors:** Zbigniew Sroka, Karina Kapusta, Senal D. Liyanage, Ta’Miyia Tobias, Victoria Petrosyan, Wojciech Kolodziejczyk, Karolina Imiełowska, Michał Gleńsk, Beata Żbikowska, Andrzej Gamian, Kamil Wojciechowski

**Affiliations:** 1 Department of Pharmacognosy and Herbal Medicines, Wroclaw Medical University, Borowska 211a, 50-556 Wroclaw, Poland; michal.glensk@umw.edu.pl (M.G.); beata.zbikowska@umw.edu.pl (B.Ż.); 2 Department of Chemistry, Physics and Atmospheric Sciences, Jackson State University, Jackson, MS 39217, USA; karina.kapusta@icnanotox.org (K.K.); kolodziejczyk.wojciech@gmail.com (W.K.); 3 Department of Chemistry and Physics, Tougaloo College, Tougaloo, MS 39174, USA; sliyanage@tougaloo.edu (S.D.L.); ttobias@student.tougaloo.edu (T.T.); victorialanapetrosyan@gmail.com (V.P.); 4 St. Andrew’s Episcopal School, Ridgeland, MS 39157, USA; 5 Faculty of Pharmacy, Wroclaw Medical University, 50-556 Wroclaw, Poland; karolina.imielowska4@gmail.com; 6 Department of Immunology of Infectious Diseases, Hirszfeld Institute of Immunology and Experimental Therapy, Polish Academy of Sciences, Rudolfa Weigla 12, 53-114 Wroclaw, Poland; andrzej.gamian@hirszfeld.pl; 7 Department of Chemistry, University of Warmia and Mazury in Olsztyn, 10-721 Olsztyn, Poland; kamil.wojciechowski@pw.edu.pl

**Keywords:** pancreatic lipase, saponin-rich plant extracts, *Panax ginseng*, *Aesculus hippocastanum*, interfacial activity, surface rheology, lipid digestion, molecular modeling, molecular dynamics, bile salt substitutes

## Abstract

Pancreatic lipase activity depends strongly on interfacial conditions created by bile salts, motivating the search for plant-derived surfactants that may modulate lipid digestion. In our previous work, ginseng root and horse chestnut seed extracts were identified as potent stimulators of pancreatic lipase. Here, we expanded this investigation to additional *Panax* and *Aesculus* species and to saponin-rich extracts from *Acer pseudoplatanus*, *Herniaria glabra*, and *Polypodium vulgare*. Extracts were evaluated for effects on pancreatin lipolysis in relation to equilibrium surface tension, surface rheology, and olive-oil emulsion stability. Antiradical and antioxidant activities, total phenolic content, and flavonoid content were also determined. White ginseng root extract (*Panax ginseng*) produced the strongest stimulation, followed by horse chestnut seed extract (*Aesculus hippocastanum*), whereas *Aesculus marylandica* seed peel extract inhibited lipase activity. Several extracts showed concentration-dependent switching from weak inhibition to stimulation. Antioxidant and antiradical activities correlated strongly with total phenolic content, while emulsion stabilization did not correlate with lipase stimulation. Molecular modeling showed that cholate, escinescin Ia, osladin, and ginsenoside Rg1 did not persistently occlude the catalytic pocket, whereas several other saponins showed active-site blocking. Together, the results support a mechanism in which saponin-rich extracts regulate lipolysis through combined interfacial effects and compound-specific enzyme interactions.

## 1. Introduction

Pancreatic lipase is the principal enzyme in fat digestion, responsible for triglyceride hydrolysis in the small intestine. It functions most efficiently near neutral pH at the lipid–water interface in the presence of colipase and bile salts [1]. Bile salts promote lipid emulsification, increase the interfacial area available for enzymatic hydrolysis, and facilitate the formation of mixed micelles that transport fatty acids and monoacylglycerols to the intestinal epithelium for absorption. Fat digestion may be impaired by pancreatic exocrine insufficiency or reduced bile salt synthesis and secretion. These conditions can occur in chronic pancreatitis, pancreatic duct obstruction, liver disease, cholestasis, and biliary obstruction and may result in steatorrhea, malnutrition, and deficiencies of fat-soluble vitamins [2,3,4,5,6,7,8,9,10]. Because bile salts are essential for lipid emulsification and efficient pancreatic lipase activity, identifying surface-active compounds that may partially support lipolysis under conditions of limited bile availability is of considerable interest.

In this work, we investigated the potential of saponins to stimulate lipolysis under conditions in which the process may be limited by insufficient bile salt activity. Saponins are structurally diverse plant secondary metabolites found in numerous medicinal and edible plants, including ginseng, horse chestnut, fenugreek, licorice, common polypody, rupturewort, and sycamore maple [11]. These compounds are plant secondary metabolites with high surface activity [12] and typically high molecular weight. Structurally, they are glycosides consisting of a sugar and a non-sugar part called an aglycone. Due to their amphipathic structure, saponins act as surfactants, interact with cell membranes, and bind substances such as phospholipids and cholesterol, thereby conferring hemolytic properties [13]. They exhibit numerous pharmacological properties, including anti-inflammatory [14], expectorant [15], antibacterial [16], and antifungal [17] activities. With respect to pancreatic lipase modulation, saponins are most commonly reported as inhibitors. Herrera et al. [18] reported on an inhibitory effect of saponin-rich extracts of fenugreek (steroid saponins) and quinoa (triterpenoid saponins) on pancreatic lipase, although the authors stressed that other components of the extracts (e.g., phenolics) could also be responsible for the inhibitory effects. A similar inhibitory effect was observed for two sulfated triterpene saponins from *Pearsonothuria graeffei* [19], for an ursolic acid stearoyl glucoside isolated from *Lantana camara* L. [20], or for a saponin-rich lyophilized juice of *Stellaria media* (Linn.) Vill. [21]. In the study by Vinarova et al., no effect on lipid digestion in the presence of diverse saponin extracts was observed [22]. Reports of pancreatic lipase stimulation are less common but include saponin-rich alfalfa extracts [23]. These contrasting findings indicate that the effects of saponins on lipolysis depend on their molecular structure, concentration, surface activity, and the overall composition of the plant extract.

In our previous work [24], we investigated the effects of nine saponin-rich plant extracts, including *Ruscus aculeatus*, *Quillaja saponaria*, *Gypsophila paniculata*, *Panax ginseng*, *Glycyrrhiza glabra*, *Primula veris*, *Hedera helix*, *Aesculus hippocastanum*, and *Trigonella foenum-graecum*, using sodium cholate as a positive control and evaluated their effects on pancreatic lipase activity in relation to their surface properties. Sodium cholate consistently produced the greatest stimulation of lipase activity, followed by *Aesculus hippocastanum* seed extract and *Panax ginseng* root extract. Molecular modeling was also performed for sodium cholate, escinescin Ia as a representative component of a lipase-stimulating extract, and hederacoside C as a representative component of a lipase-inhibiting extract. These results provided initial evidence that the effects of saponin-rich extracts on lipolysis may arise from a combination of interfacial properties and compound-specific interactions with pancreatic lipase. However, the limited number of modeled saponins and plant extracts did not allow us to determine whether this proposed mechanism could be generalized across structurally diverse saponins or closely related plant species.

Building upon these findings, the present study aims to determine whether the contrasting stimulatory and inhibitory effects of saponin-rich extracts can be explained by a broader relationship among their phytochemical composition, interfacial behavior, and interactions of individual saponins with pancreatic lipase. The investigation was therefore expanded to additional *Panax* species (*Panax ginseng* white root, *Panax ginseng* red root, *Panax quinquefolius*, and *Panax notoginseng*) and *Aesculus marylandica* alongside *Aesculus hippocastanum*. We investigated several additional saponin-rich plants, including *Acer pseudoplatanus*, *Herniaria glabra*, and *Polypodium vulgare*, to broaden the structural and phytochemical diversity of the investigated extracts. More importantly, this study goes beyond evaluating lipase stimulation alone by integrating phytochemical characterization (UHPLC-MS), antioxidant and antiradical analyses, surface tension and rheological measurements, emulsion stability studies, and molecular dynamics simulations for a variety of saponins. This integrated experimental and computational approach enables a substantially deeper mechanistic understanding of how saponin-rich extracts influence pancreatic lipase through both interfacial effects and direct interactions with the enzyme.

## 2. Results

### 2.1. The Influence of Saponin-Rich Extracts on the Lipolytic Activity of Pancreatin

Lipases are enzymes that digest substrates that are insoluble in water (lipids), so their activity strongly depends on the presence of surfactants in the reaction mixture. All extracts tested in this study are rich in saponins, i.e., natural surfactants, so we assumed that these extracts could have a beneficial effect on lipase activity. Our test conditions largely reflect those under which pancreatic lipase operates in the duodenum.

**Figure 1:**
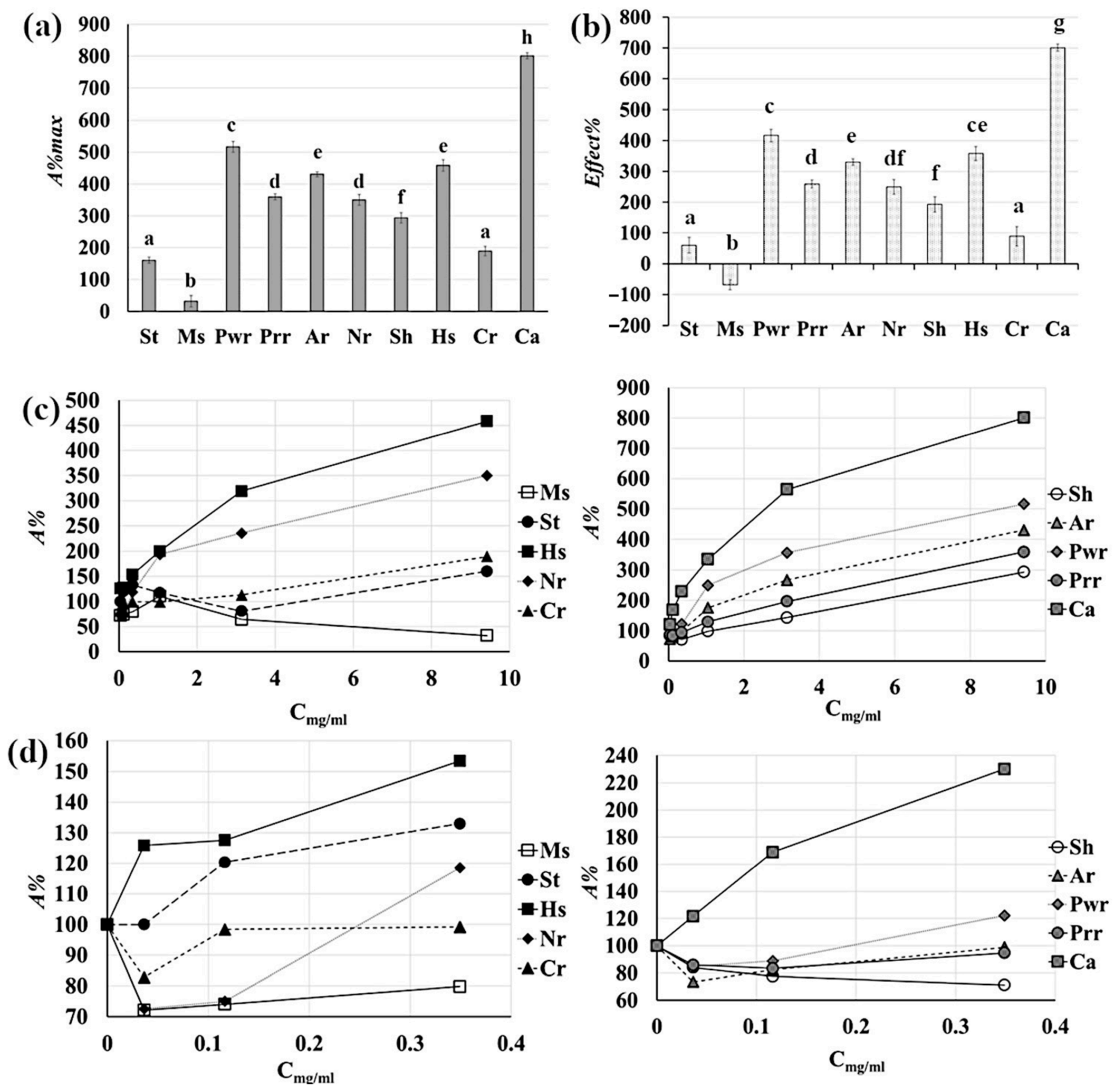
Lipolytic activity of pancreatin: (**a**) Relative lipase-stimulating activity of the sample compared to the control test (without extract), *A%max*; (**b**) Difference between the lipase activity at the maximum concentration of extract (9.45 mg/mL in the reaction mixture) and the sample without extract (*Effect%*). The effect of the extract on lipase activity at each extract concentration expressed as a percentage in the (**c**) full extract concentration range of 0–9.45 mg/mL in the reaction mixture and (**d**) in the low extract concentration range of 0–0.35 mg/mL in the reaction mixture. Extract names: *Acer pseudoplatanus* (twigs)—**St**, *Aesculus marylandica* (seed peels)—**Ms**, *Panax ginseng* (white root)—**Pwr**, *Panax ginseng* (red root)—**Prr**, *Panax quinquefolius* (root)*—***Ar**, *Panax notoginseng* (root)*—***Nr**, *Herniaria glabra* (herb)*—***Sh**, *Aesculus hippocastanum* (seeds)—**Hs**, *Polypodium vulgare* (rhizome)—**Cr**, sodium cholate—**Ca**; *n* = 3. There is no statistical significance of differences between values marked with the same letter.

The results are demonstrated as the effect of the extract on lipase activity at each extract concentration expressed as a percentage (*A%*), the relative lipase-stimulating activity of the sample compared to the control test without extract (*A%max*), and the difference between the lipase activity at the maximum concentration of extract and the sample without extract in relation to the sample without extract (*Effect%*) in Table 1 and Figure 1. The highest stimulation of lipase activity was observed for sodium cholate (**Ca**), where *A%max* and *Effect%* were equal to 801 ± 10 and 701 ± 11. This observation in both our previous study [24] and the present investigation further supports its use as a reliable positive control. Then, in decreasing order, values of *A%max* and *Effect%* were equal to **Pwr** (517 ± 17, 417 ± 21) > **Hs** (459 ± 18, 359 ± 23) > **Ar** (431 ± 8, 331 ± 10) > **Prr** (360 ± 9, 260 ± 13) > **Nr** (350 ± 17, 250 ± 24) > **Sh** (294 ± 16, 194 ± 25) > **Cr** (189 ± 15, 89 ± 31) > **St** (160 ± 10, 60.2 ± 25.9) > **Ms** (31.7 ± 18.0, −68.3 ± 16.3). Figure 1c,d show the effects of extracts at different concentrations on lipase activity. The highest stimulating effect was observed in the presence of sodium cholate. The second lipase-stimulating effect was found for the white ginseng root extract (**Pwr**), and the third for the horse chestnut seed extract (**Hs**). An interesting observation is that, at low concentrations, the horse chestnut seed extract (Figure 1c) immediately exhibits a stimulating effect, acting similarly to sodium cholate, without the initial inhibition observed with other extracts. In the case of white ginseng root extract, as well as other extracts at the lowest concentrations, an inhibitory effect on lipase activity was first observed; at higher concentrations, stimulation of lipase was noted.

### 2.2. Antiradical and Antioxidant Activity of Extracts

**Figure 2:**
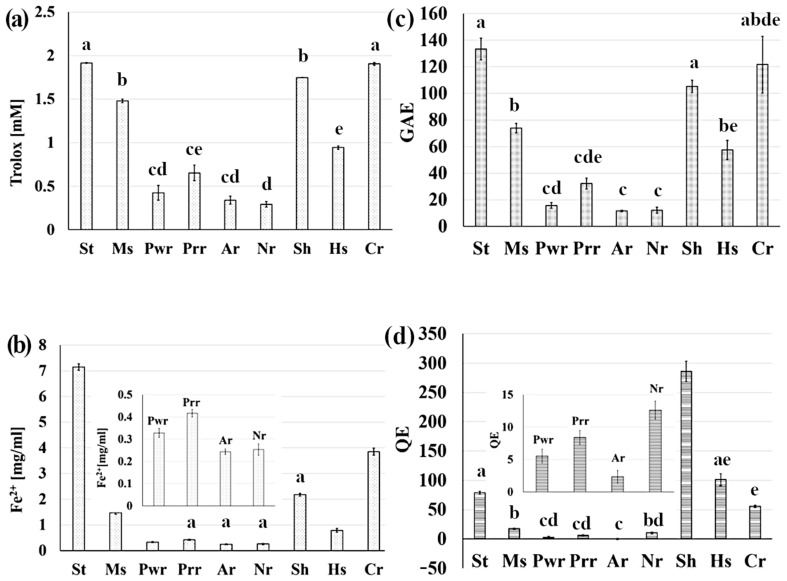
Antioxidant and antiradical activity of extracts, demonstrated as: (**a**) the ability of extracts to quench the ABTS^•+^ cation radical, shown as Trolox equivalents Trolox [mM]; (**b**) the ability of extracts to reduce Fe^+3^ ions to Fe^+2^, shown as the amount of Fe^+2^ [mg/mL] (activities of less active extracts are zoomed); (**c**) the total amount of phenolic compounds demonstrated as gallic acid equivalents (GAE); and (**d**) the amount of flavonoids, expressed as quercetin equivalents (**QE**) (extracts with a lower amount of flavonoids are zoomed)**.** Extracts: *Acer pseudoplatanus* (twigs)—**St**, *Aesculus marylandica* (seed peels)—**Ms**, *Panax ginseng* (white root)—**Pwr**, *Panax ginseng* (red root)—**Prr**, *Panax quinquefolius* (root)*—***Ar**, *Panax notoginseng* (root)*—***Nr**, *Herniaria glabra* (herb)*—***Sh**, *Aesculus hippocastanum* (seeds)—**Hs**, *Polypodium vulgare* (rhizome)—**Cr**. There is no statistical significance of differences between values marked with the same letter.

Antiradical activity, measured using the ABTS^•+^ radical, is demonstrated as Trolox equivalents in Table 2 and Figure 2a. Extract **St** (1.92 ± 0.02, Trolox [mM]) showed the greatest antiradical activity. Other extracts showed the following activities in decreasing order: **Cr** (1.90 ± 0.01, Trolox [mM]) > **Sh** (1.75 ± 0.01, Trolox [mM]) > **Ms** (1.48 ± 0.07, Trolox [mM]) > **Hs** (0.94 ± 0.05, Trolox [mM]) > **Prr** (0.65 ± 0.06, Trolox [mM]) > **Pwr** (0.42 ± 0.03, Trolox [mM]) > **Ar** (0.33 ± 0.05, Trolox [mM]). The lowest antiradical activity was observed for extract **Nr** (0.29 ± 0.04 Trolox [mM]).

The reducing activity of the extracts was measured by the reduction of Fe^3+^ to Fe^2+^ and expressed as millimoles of Fe^2+^ produced per gram of extract, as shown in Table 2 and Figure 2b. The strongest activity was noted for extract **St** (7.20 ± 0.12, Fe^2+^ [mg/mL]), followed by the other extracts in decreasing order: **Cr** > **Sh** > **Ms** > **Hs** > **Prr** > **Pwr** > **Nr** (0.25 ± 0.03, Fe^2+^ [mg/mL]) ≈ **Ar** (0.24 ± 0.01, Fe^2+^ [mg/mL]).

### 2.3. Amount of Total Phenolics and Flavonoids in Extracts

The amount of total phenols and flavonoids in the extracts is shown in Table 2 and Figure 2c,d. The highest amount of total phenols was found in the **St** (133.3 ± 8.1 GAE ± SD) extract; a smaller amount of phenols was measured in **Cr** (121.7 ± 21.2) > **Sh** (105.3 ± 4.7), followed in decreasing order by **Ms** > **Hs** > **Prr** > **Pwr** > **Nr**; and the lowest amount was found in **Ar** (11.7 ± 0.59 GAE ± SD). The highest amount of flavonoids was found in extract **Sh** (284.70 ± 17.40 QE ± SD), followed by **Hs** (102.13 ± 10.28) and **St** (80.24 ± 2.54). The least amount of flavonoids was found in the **Ar** extract (2.35 ± 0.98 QE ± SD).

### 2.4. UHPLC Analyses of the Plant Extracts

**Figure 3:**
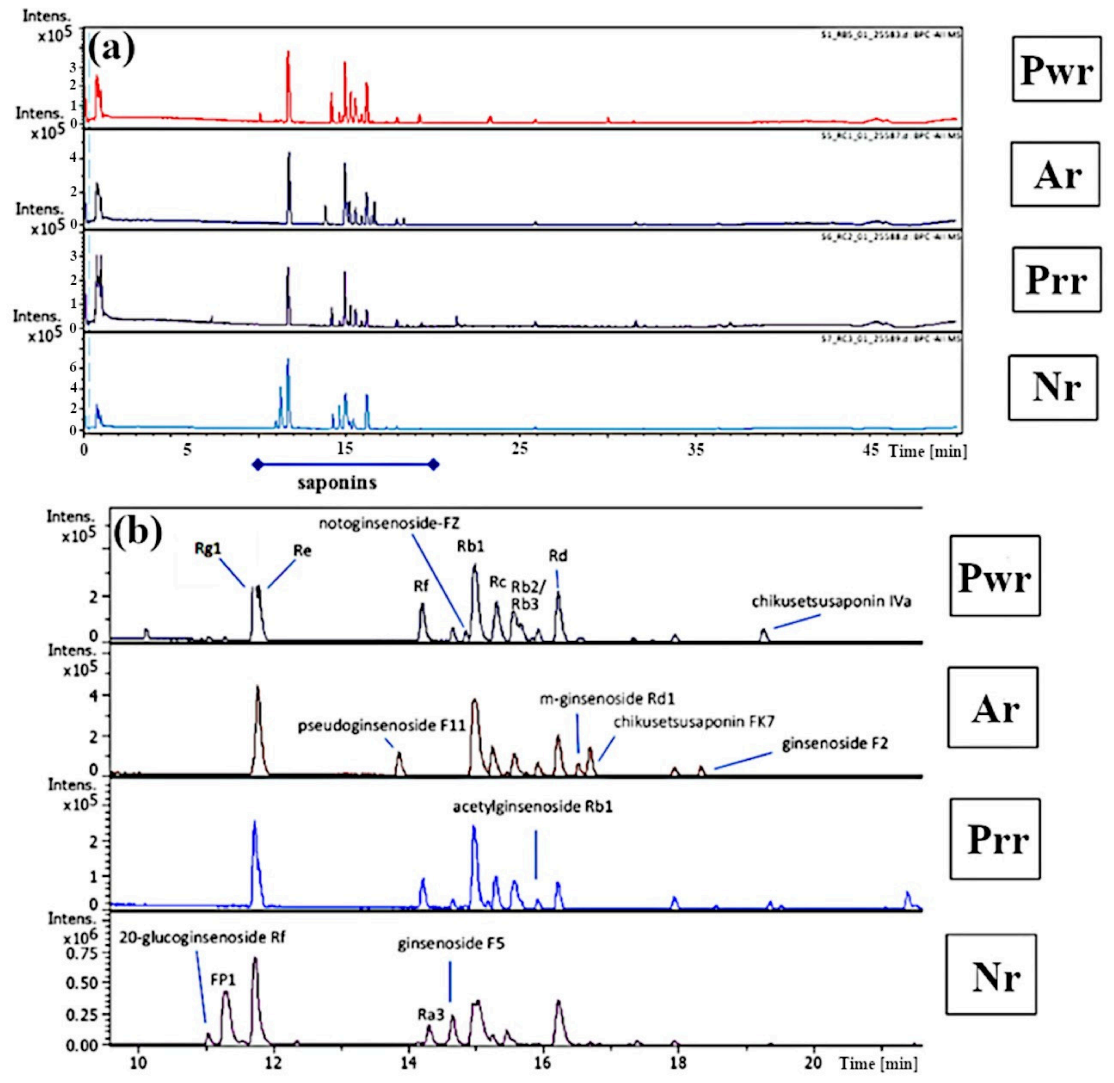
(**a**) UHPLC-MS base peak ion (BPI) chromatograms of the investigated *Panax* species extracts; (**b**) LC-MS base peak ion (BPI) chromatograms (selected range 10–20 min) of the investigated *Panax* species extracts. Extracts: *Panax ginseng* (white root)—**Pwr**, *Panax ginseng* (red root)—**Prr**, *Panax quinquefolius* (root)*—***Ar**, *Panax notoginseng* (root)*—***Nr**.

The most active extracts with the highest stimulatory effect on the lipolytic activity of pancreatin were extracts from four ginseng roots. These four extracts were analyzed by LC-MS to characterize and compare their phytoconstituents. The data are presented in Figure 3 and Table 3. The most abundant saponin peaks were tentatively characterized based on their MS and MS/MS spectra and were in good agreement with those previously reported in the literature [25,26,27].

### 2.5. Surface Properties of Tested Extracts

The equilibrium surface tension, γ_eq_, for 1% solutions of the nine saponin-rich extracts tested for their effect on pancreatin activity is reported in Table 4. The values vary over a wide range between 33.7 mN/m (for **Pwr**) and 58.4 mN/m (for **St**). In terms of the surface compression rheological parameters of the adsorbed layers formed on the surface of 1% extract solutions, **Ms** clearly stands out with its storage modulus, E’ = 188 mN/m (Table 4), while all other extracts showed moderate values of E’, in the range of 7.6 mN/m–32.5 mN/m. In terms of surface viscous behavior, only two extracts showed loss moduli, E”, in excess of 14 mN/m (**Ms** and **Hs**). For all other adsorbed layers, the liquid-like response was negligible.

The ability of the investigated extracts to reduce surface tension may change in the presence of pancreatin, even though the latter has been shown to be only weakly surface-active at the water–air interface (γ_eq_ = 50.9 mN/m). To assess the effect of the enzyme, surface tension and surface compression rheology parameters were determined for 1:1 (*w*/*w*) mixtures of pancreatin with the extracts. For **Ms**, **Sh**, **Prr**, and **St**, the presence of pancreatin further reduced their surface tension, whereas the opposite effect was observed for the other extracts. In the case of **Cr**, the reduced surface activity is likely due to the precipitation of an insoluble product from a reaction between the enzyme and the extracted components. As a result, at least part of the surface-active components might have been removed from the solution, as further corroborated by the reduced surface compression rheological parameters (Table 4).

**Figure 4:**
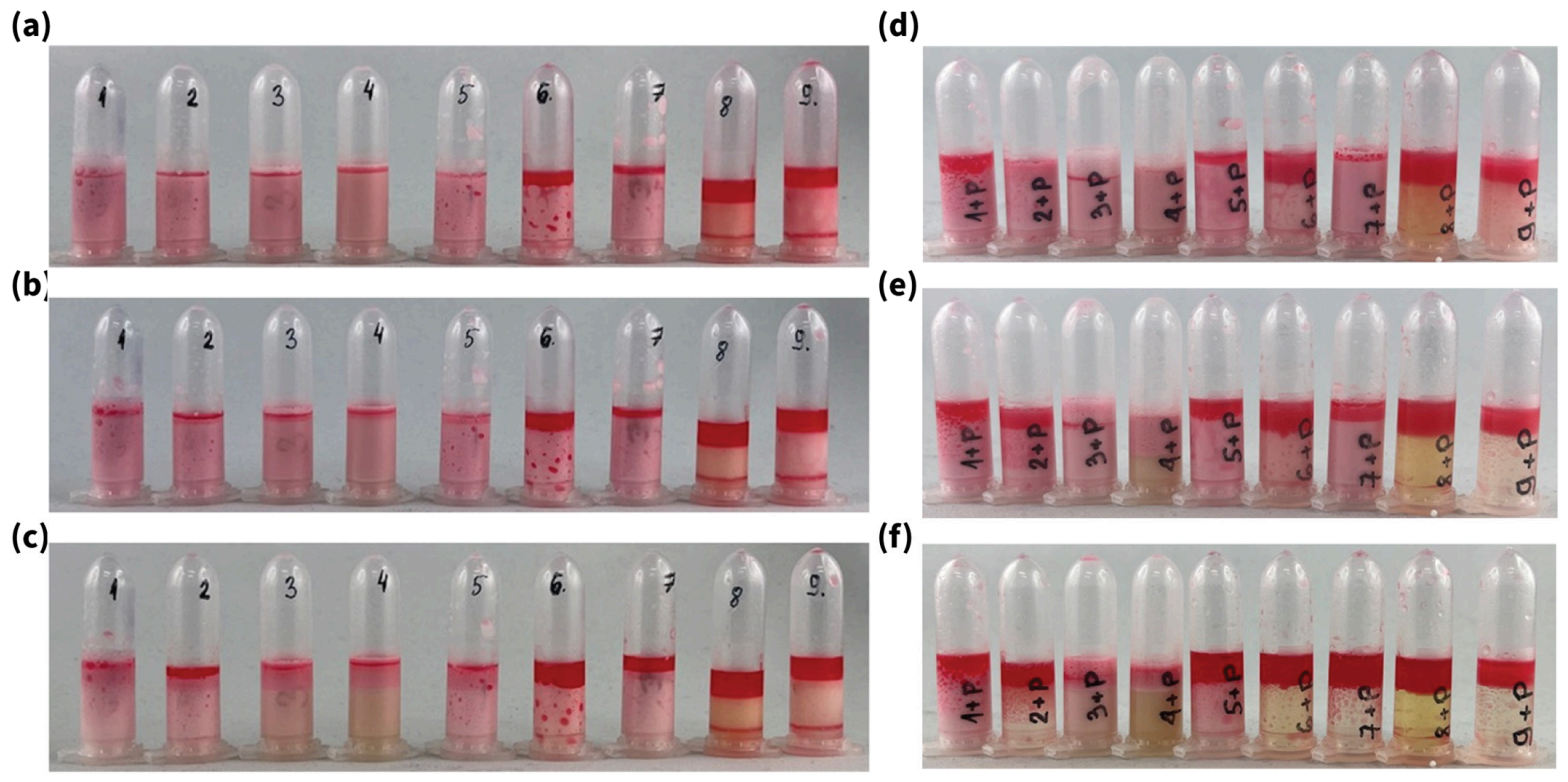
Photographs of emulsions prepared from the mixtures containing 1% extract as an aqueous phase homogenized with olive oil (stained with Sudan Red IV) immediately after homogenization (**a**), after 1 h (**b**), and after 24 h (**c**) and the mixtures containing 1% extract and 1% pancreatin as an aqueous phase homogenized with olive oil (stained with Sudan Red IV) immediately after homogenization (**d**), after 1 h (**e**), and after 24 h (**f**). The extracts used as emulsifiers (from left): 1—*Aesculus hippocastanum* (seeds) **Hs**, 2—*Panax ginseng* white (root) **Pwr**, 3—*Aesculus marylandica* (seed peels) **Ms**, 4—*Herniaria glabra* (herb) **Sh**, 5—*Panax quinquefolius* (root) **Ar**, 6—*Panax ginseng* red (root) *radix* **Prr**, 7—*Panax notoginseng* (root) **Nr**, 8—*Polypodium vulgare* (rhizome) **Cr**, and 9—*Acer pseudoplatanus* (twigs) **St**.

To enable a comparison of the emulsification capabilities of the investigated extracts and their 1:1 mixtures with pancreatin, two series of emulsions were prepared in olive oil under identical conditions (Figure 4). For the individual extracts and solutions, the first clear signs of demulsification appeared immediately after homogenization, especially in **Prr**, **Cr**, and **St**, where a red oil layer separated at the top (Figure 4d–f). The situation did not change much within 1 h, but after 24 h, a clearly demulsified oil layer appeared additionally in **Nr**. The remaining extracts were capable of stabilizing the model emulsions reasonably well, even 24 h after homogenization, with most of the oil remaining in an emulsified form (Figure 4a–c). For the mixtures containing pancreatin and the **Prr**, **Cr**, and **St** extracts, the oil phase separated immediately after homogenization was stopped, similar to the corresponding emulsions without pancreatin (Figure 4d–f). Nevertheless, while in the latter some emulsion phase remained after 24 h, it disappeared completely within 1 h (for **Cr** and **St**) or 24 h (for **Prr**) in the presence of the enzyme. Only two extracts were able to maintain the emulsion without phase-separating the oil phase within 24 h: **Sh** and **Ms**, regardless of the presence of pancreatin and the resulting change in surface tension.

### 2.6. Modulation of Pancreatic Lipase by Saponins on the Molecular Level

**Figure 5:**
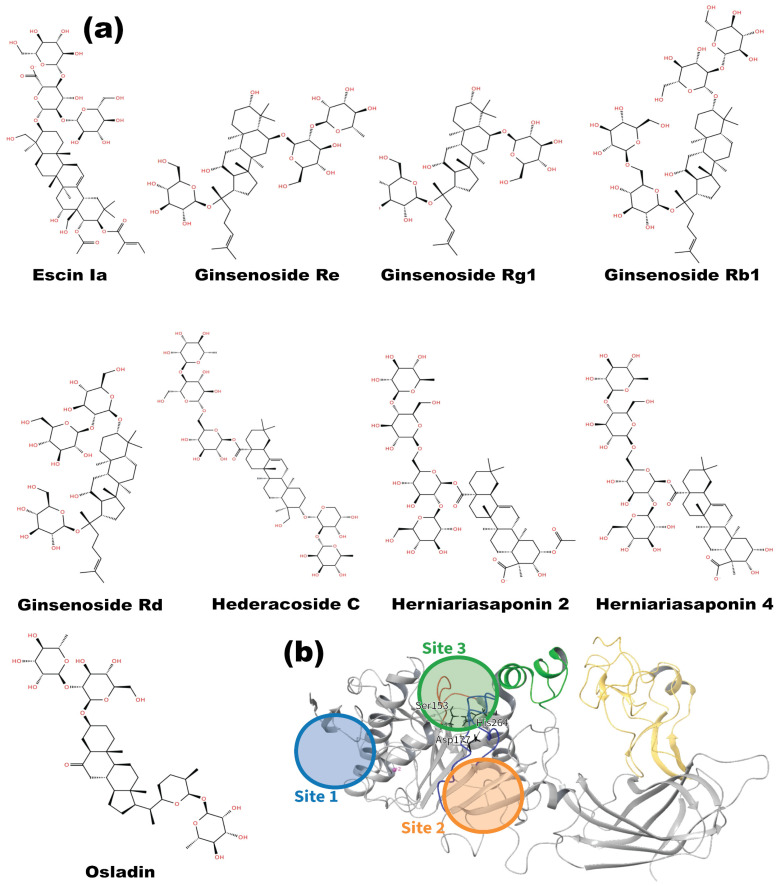
Computational model system and ligands: (**a**) 2D structures of the selected saponins and reference ligands; (**b**) porcine pancreatic lipase–colipase complex with the three investigated binding regions highlighted.

To generate molecular-level hypotheses for how representative saponin constituents may interact with pancreatic lipase, selected saponins were evaluated computationally (Figure 5a). The selected compounds included ginsenosides Rg1, Re, Rb1, and Rd from *Panax* species; escinescin Ia from *Aesculus hippocastanum*; herniariasaponins 2 and 4 from *Herniaria glabra*; and osladin from *Polypodium vulgare*. Cholate anion was included as a reference bile salt surfactant because it produced the strongest stimulation of pancreatic lipase activity. Hederacoside C was included as a reference inhibitor based on our previous work [24] as a compound that exhibited strong inhibitory activity against pancreatic lipase. In the previous work, two main glycan-binding regions on pancreatic lipase were identified, referred to here as site 1 and site 2 (Figure 5b) [24]. In addition, hederacoside C was shown to display high binding affinity toward the catalytic active-site region, referred to here as site 3 (Figure 5b). Therefore, in the present study, we evaluated all three possible saponin-binding regions to determine whether the representative saponins preferentially interact with non-catalytic surface sites or with the active-site region of pancreatic lipase. This distinction is mechanistically important because pancreatic lipase activity is controlled not only by catalytic-site access but also by the geometry of the lid, β5 loop, β9 loop, colipase-supported interfacial binding, and surfactant-mediated substrate presentation. Therefore, ligands that do not persistently occupy the catalytic pocket may still influence lipolysis by altering substrate-entry geometry or the dynamics of interfacial activation-related regions.

**Figure 6:**
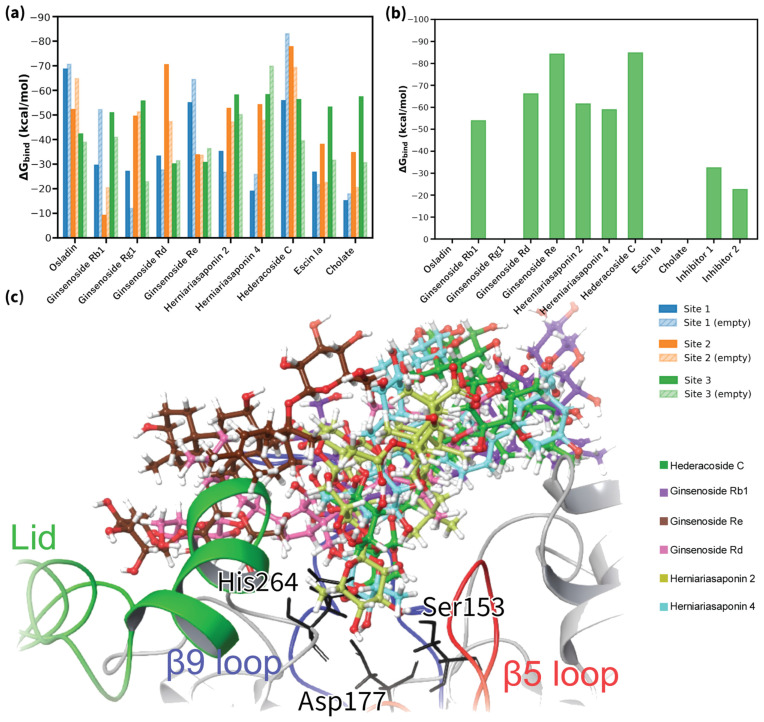
Docking/MM-GBSA results and representative post-MD binding modes: (**a**) initial MM-GBSA scores for ligand binding to the empty receptor and to the receptor with other binding sites occupied; (**b**) MM-GBSA scores recalculated for representative clustered MD structures; (**c**) representative 3D binding modes from the most populated MD clusters.

We first performed consecutive docking, in which each ligand was docked sequentially into sites 1, 2, and 3. Although site 3 was centered on the catalytic triad of pancreatic lipase, composed of Ser153, Asp177, and His264, docking of osladin, escin Ia, and cholate resulted in binding modes distinct from those observed for the other saponins. Instead of penetrating the catalytic pocket, osladin, escin Ia, and cholate were positioned near the substrate-entry region, particularly around the lid domain and the β5 loop. Similarly, ginsenoside Rg1 was predicted to bind near the lid/β9-loop region without direct occupation of the catalytic pocket. In contrast, several other saponins adopted poses within or near the active-site entrance. These results suggest that the ligands differ less by simple active-site versus non-active-site binding and more by the extent to which they approach the substrate-entry gateway formed by the lid, β5 loop, β9 loop, and catalytic-site region. Molecular mechanics generalized Born surface area (MM-GBSA) calculations were performed to refine the binding modes while allowing partial flexibility of the protein near the binding site. Binding free energy values were calculated in two scenarios: binding of the docked ligand to the empty protein and binding when other ligands were already occupying the remaining binding sites (Figure 6a). The results varied across the three sites. For active-site binding, most ligands showed stronger predicted binding affinity when the other binding sites were already occupied, except for ginsenosides Re and Rd and herniariasaponin 4, which exhibited lower binding affinity in the presence of other ligands. The highest overall binding affinity was observed for hederacoside C, which bound strongly to all three binding sites, with particularly high affinity at site 2. Osladin showed the strongest affinity for site 1, while several saponins showed favorable binding within the active-site region. Because MM-GBSA scoring does not necessarily reflect the long-term stability of protein–ligand complexes, refined structures with all three ligand sites occupied were subjected to molecular dynamics simulations to evaluate complex stability and structural changes within the enzyme.

Molecular dynamics simulations yielded equilibrated protein structures across all complexes. Protein root mean square deviation (RMSD) values did not exceed 2 Å after rapid stabilization, and no major global conformational changes were observed during the 500 ns simulations (Supporting Information, Figure S1). The highest protein root mean square fluctuations (RMSF) were observed in the lid region (Supporting Information, Figure S2), particularly in complexes with herniariasaponin 4 and ginsenoside Rg1, followed by osladin and hederacoside C. Lower lid-region fluctuations were observed for ginsenosides Re and Rd. Osladin, ginsenoside Rb1, escinescin Ia, and cholate did not block the active-site region throughout the simulations, whereas the remaining ligands showed stable binding within or near the active site.

For the most populated clusters from the MD trajectories, MM-GBSA calculations were repeated to account for changes in complex geometry during simulation (Figure 6b). The strongest binding affinities were observed for hederacoside C and ginsenoside Re. Notably, all saponins predicted to bind within the active site exhibited lower binding free energy values than the co-crystallized inhibitor from the 1ETH structure of the porcine lipase–colipase complex [28]. The saponins differed not only in binding affinity but also in their specific binding modes. The ginsenosides did not penetrate deeply into the active-site binding pocket; instead, they bound to the region between the lid and the β9 loop (Figure 6c). In the cases of ginsenosides Re and Rd, ligands initially bound to sites 1 and 2 relocated and eventually occupied the active-site region, interacting with the lid and β9 loop. Both compounds formed strong interactions with Cys238, Gln245, and Asn263, which persisted for over 80% of the final 50 ns of the simulation. The interaction with Gln245 persisted for over 80% of the full 500 ns trajectory (Supporting Information, Figure S3). Although ginsenoside Rb1 maintained fewer persistent contacts than Re or Rd, its active-site-associated pose remained near the β9-loop/active-site region during the final part of the simulation (Supporting Information, Figure S4). Ginsenoside Rb1 interacted through its sugar ring with Asp206, a residue located in the β9 loop near the active-site triad.

**Figure 7:**
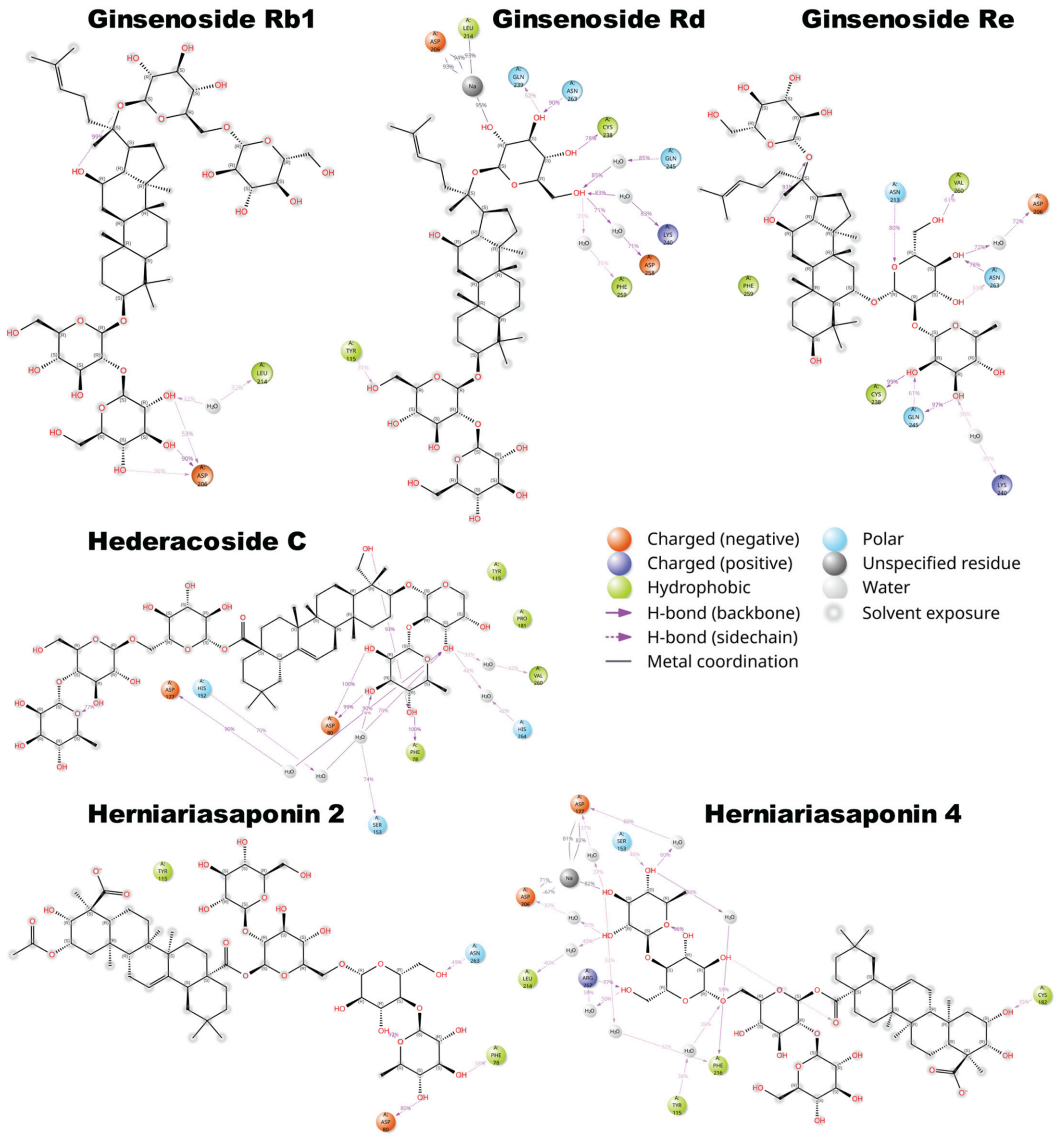
2D interaction diagrams for saponins bound within the active site.

The larger saponins, herniariasaponin 2, herniariasaponin 4, and hederacoside C, had one of their sugar rings oriented toward the active-site residues. The relocation observed for ginsenosides Re and Rd was not observed in these cases. Hederacoside C was highly solvent-exposed and flexible when bound outside the active-site region. Within the active site, interactions were observed with the catalytic triad residues Ser153, Asp177, and His264, primarily through hydrogen bonds and water bridges. Herniariasaponin 2 also showed high ligand flexibility outside the active site, whereas herniariasaponin 4 behaved more similarly to ginsenosides Rd and Re by forming a cluster near the active site. Both herniariasaponins interacted with the catalytic triad or nearby residues. Ligand–protein contacts for ligands bound within the active-site region are shown in Figure 7.

Escin Ia remained comparatively more stable at sites 1 and 2 but showed substantial mobility from its initial pose at site 3, consistent with relocation away from persistent catalytic pocket occlusion as reflected by its higher ligand RMSD and RMSF values after fitting to the protein (Supporting Information, Figures S4 and S5). In site 1, escinescin Ia interacted with Arg164 for 64% of the simulation time; in site 2, it interacted with Gln184 for 33% of the simulation time; and after relocation from the original site 3 position, it interacted with Leu36 and Arg38. Ginsenoside Rg1 showed stable binding within site 1 but substantial relocation within sites 2 and 3. In site 1, it was stabilized by calcium-mediated coordination involving Arg191, Asp193, Glu188, Leu189, and Gly160. Strong hydrogen bonds were also formed with Asp196 and Ser195. Binding within sites 2 and 3 was less stable. Osladin also showed relocation within sites 2 and 3, with no persistent binding observed in site 3 and high ligand fluctuations. In site 2, osladin formed a hydrogen bond with Val322 for 71% of the simulation time. In site 1, where it was more stable, osladin formed two hydrogen bonds with Asp287, each persisting for approximately 35–39% of the simulation time. Finally, cholate was largely solvent-exposed in sites 2 and 3 and showed high overall fluctuations. At the beginning of the trajectory, cholate transiently formed a hydrogen bond with Arg123, but it relocated after approximately 300 ns.

**Figure 8:**
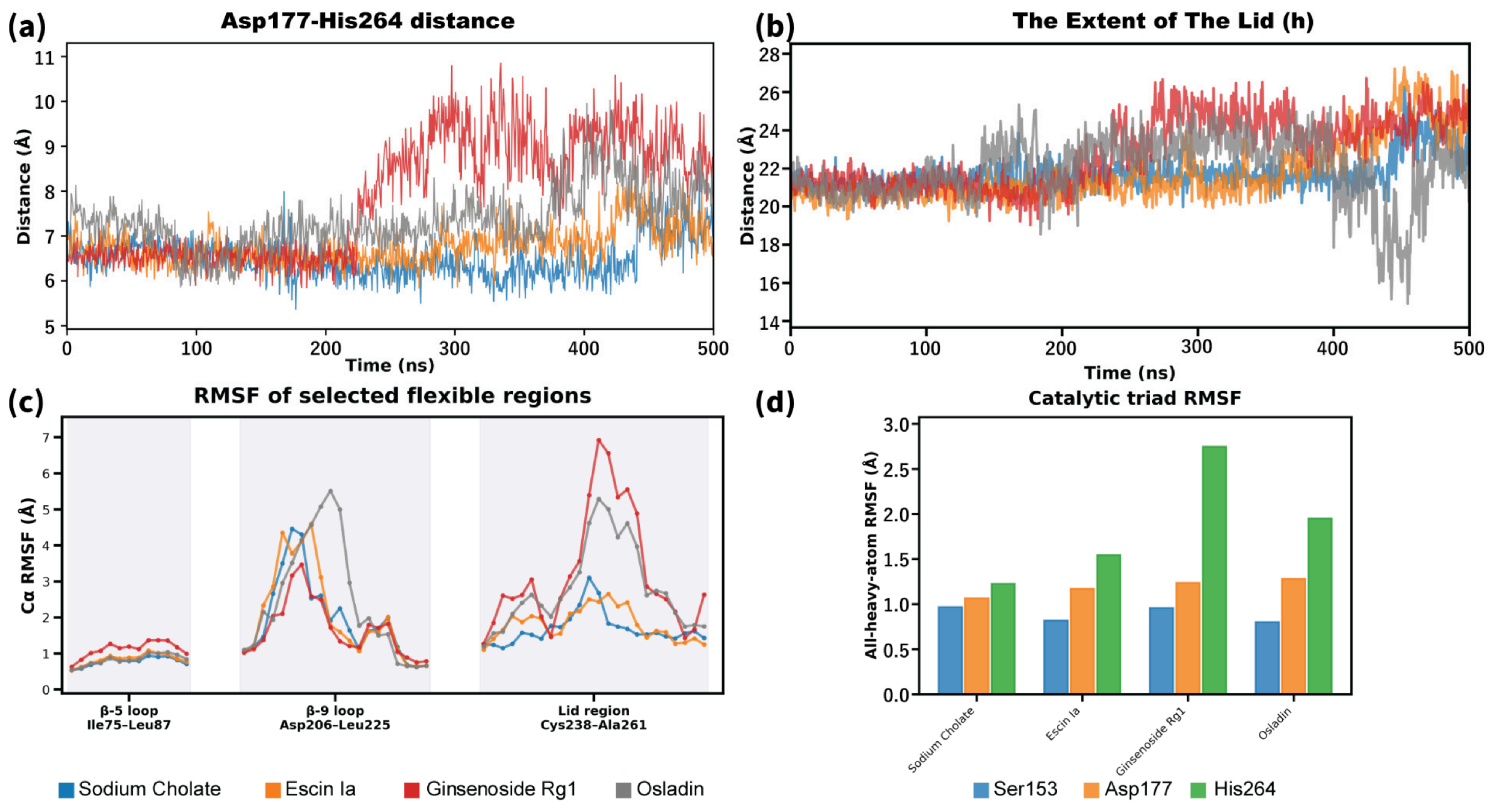
Ligand-dependent structural dynamics of the active site and flexible regions. Time-dependent distances between catalytic/structural residues for selected ligand-bound systems: (**a**) Asp177–His264 distance between catalytic triad residues (measured between Cα) and (**b**) the extent of the lid represented as a height (h) of the triangle between the tips of the gateway loops β-5, β-9, and the lid (measurement was made between Cα of PHE 78, PHE 216, and ILE 252, respectively, pointed from ILE 252); (**c**) RMSF analysis of selected flexible regions, including the β-5 loop (Ile75–Leu87), β-9 loop (Asp206–Leu225), and lid region (Cys238–Ala261); (**d**) all-heavy-atom RMSF values of the catalytic triad residues Ser153, Asp177, and His264.

While binding within the active-site region may suggest a competitive inhibition mechanism, ligand binding outside the active site can also affect enzyme activity by altering protein dynamics. To investigate this possibility, additional structural analyses were performed on systems containing osladin, ginsenoside Rg1, and escinescin Ia, and the results were compared with those for the cholate anion complex. These analyses focused on residue–residue distances and RMSF changes in functionally important regions. In our previous work, cholate and escinescin Ia were associated with shorter Asp177–His264 distances than hederacoside C, which was interpreted as part of an activator-like structural response. In the present analysis, the Asp177–His264 distance remained relatively stable for cholate and escinescin Ia throughout most of the 500 ns simulation (Figure 8a). In contrast, ginsenoside Rg1 showed a clear increase in this distance after approximately 220 ns. Osladin displayed intermediate behavior, with fluctuations becoming more pronounced toward the later part of the simulation. During the final 50 ns, all systems showed slightly increased Asp177–His264 distances, with cholate and escin Ia maintaining the lowest values and ginsenoside Rg1 showing the highest values. The lid-height analysis also showed ligand-dependent differences (Figure 8b). Cholate and escin Ia showed gradual increases in lid height over time. Ginsenoside Rg1 showed a more pronounced increase beginning around 240–260 ns. Osladin displayed greater variability, including a noticeable decrease in lid height between approximately 400 and 460 ns, followed by partial recovery toward the end of the simulation. RMSF analysis of selected flexible regions showed that the β5 loop remained relatively stable across all systems, with slightly higher fluctuations observed for ginsenoside Rg1 (Figure 8c). Larger ligand-dependent differences were observed in the β9 loop and lid region. Ginsenoside Rg1 produced the highest RMSF values in the lid region, while osladin also increased flexibility in both the β9 loop and lid region. Cholate and escin Ia generally showed lower RMSF values within the lid region. The all-heavy-atom RMSF values of the catalytic triad residues showed that His264 was the most flexible residue in all systems, with the largest increase observed for ginsenoside Rg1, whereas Ser153 was the least flexible residue (Figure 8d).

In addition to molecular modeling approaches, we utilized in silico methods to predict the potential toxicity of saponins. The ProTox 3.0 web server is a reliable tool for the preliminary screening of potential toxicity flags. Because of the molecular-size limitations of the ProTox-3.0 server, toxicity predictions could be completed only for ginsenoside Rg1 among the investigated saponins. Sodium cholate was evaluated as a representative bile salt for comparison. The predicted oral LD_50_ of ginsenoside Rg1 was 4000 mg kg^−1^, corresponding to toxicity class 5, whereas sodium cholate had a predicted oral LD_50_ of 2000 mg kg^−1^ and was assigned to toxicity class 4. Sodium cholate was predicted to be active for hepatotoxicity, respiratory toxicity, cardiotoxicity, and immunotoxicity, with a lower-probability prediction for nephrotoxicity. Ginsenoside Rg1 was predicted to be active for cardiotoxicity, immunotoxicity, and nutritional toxicity.

## 3. Discussion

Previous studies, including ours, have demonstrated that certain saponins and saponin-rich extracts stimulate pancreatic lipase activity [23,24]. In particular, extracts from *Aesculus hippocastanum* seeds and *Panax* roots produced high stimulatory effects approaching approximately one-half of that observed for sodium cholate, the principal bile salt. Consistent with our previous study [24], sodium cholate again exhibited the strongest stimulation of pancreatic lipase activity (*Effect%* ≈ 701 and 958 for the current and previous study, respectively), confirming the reproducibility of this observation and supporting its use as a reliable positive control. Likewise, *Aesculus hippocastanum* remained one of the most potent lipase-stimulating extracts (*Effect%* ≈ 359 and 240 for the current and previous study, respectively). These results confirm the consistently dominant stimulatory effect of sodium cholate while demonstrating that the magnitude of the response to plant extracts may vary depending on the investigated plant material and extract composition. Among the newly investigated plant materials, white *Panax ginseng* root exhibited the greatest stimulatory effect *(Effect%* ≈ 417), outperforming not only *Aesculus hippocastanum* but also all other investigated *Panax* species. Unexpectedly, the closely related species *Aesculus marylandica* produced the opposite response, inhibiting pancreatic lipase activity. This striking contrast between two species of the same genus suggests that lipase modulation depends on the qualitative phytochemical composition of the extracts rather than on their taxonomic relationship alone. Interestingly, escin Ia, one of the major saponins identified in *Aesculus* species, did not exhibit inhibitory activity in our in silico studies, indicating that the inhibition observed for *Aesculus marylandica* is unlikely to result from a similar mechanism. Further phytochemical characterization of *Aesculus marylandica* will therefore be necessary to identify the constituents responsible for its inhibitory activity.

Extracts obtained from plant raw materials were rich in various substances, including phenols and polyphenols, which constitute a highly structurally diverse group of compounds with varied pharmacological effects [29]. This group includes flavonoids with spasmolytic [30], diuretic and choleretic effects, tannins with astringent and antihemorrhagic effects [31], and procyanidins with antiatherosclerotic activity [32].

**Figure 9:**
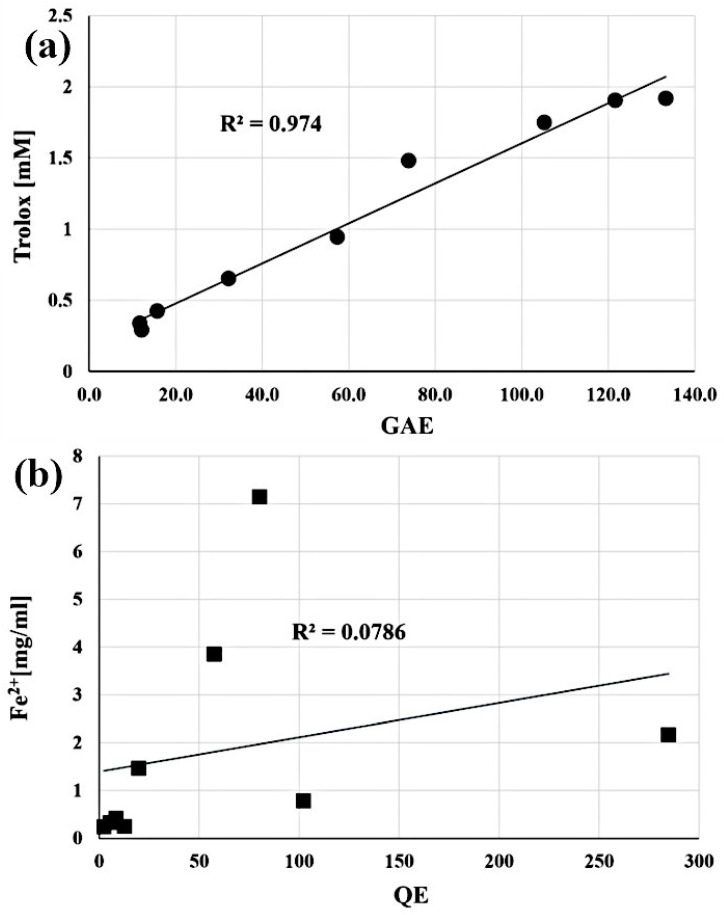
(**a**) High positive correlation between the antiradical properties of extracts (Trolox [mM]) and total phenolic content (GAE). (**b**) No correlation between the flavonoid content (QE) and the antioxidant activity of extracts (Fe^2+^ [mg/mL]).

All phenolic compounds have antioxidant and antiradical activity to a greater or lesser extent [33]. Our research confirmed the high phenolic content in the extracts and the high antiradical and antioxidant activities of some of them. The strong correlation coefficients (R) between the total phenolic content (GAE) and the antiradical activity, expressed as Trolox [mM] (Figure 9a), and the reducing activity of extracts, expressed as Fe^2+^ [mg/mL] concentration, were 0.99 and 0.87, respectively. Weak correlations (R) were observed between flavonoid content (QE) and Trolox [mM] and Fe^2+^ [mg/mL] (Figure 9b), with correlation coefficients of 0.56 and 0.28, respectively.

Extracts from natural raw materials have numerous pharmacological properties that depend on their chemical composition. Pharmacological activity is the result of the action of various compounds, mainly the dominant ones, but it also depends on the strength of the biological action of these compounds. Pharmacologically important substances include saponins, which have the following effects: expectorant [15], adaptogenic [34], anti-inflammatory [35], and the inhibition of transcapillary filtration [36]. The distinguishing feature of saponins among natural compounds is their surface activity; they are detergents [37]. These features formed the basis of the research described in this paper.

Lipases are enzymes that digest hydrophobic, water-insoluble substrates. Their activity is strongly stimulated by the presence of surface-active substances such as bile salts (e.g., sodium cholate) [38]. One of the aims of this study was to investigate whether extracts rich in surface-active saponins would be able to stimulate lipase activity, in this case, pancreatic lipase, which is a component of pancreatin. Most of the literature data describe the inhibitory effect of saponins on lipase activity [39]. There are fewer papers describing the stimulation of lipases by saponins and extracts rich in saponins. Stimulation of lipoprotein lipase (LPL) by saponins from ginseng root, with a mechanism of action similar to that of pancreatic lipase, has been observed [40]. In an earlier paper, the stimulation of pancreatic lipase in the presence of digitonin, a steroidal saponin from foxglove [41], was described.

One of the most informative surface activity parameters for comparing different (bio)surfactant solutions is surface tension. Although lipid digestion occurs at the liquid–liquid interface stabilized by (bio)surfactants (e.g., bile acids and surfactants introduced with food, such as emulsifiers), we analyzed the adsorption behavior of the plant extracts at the water–air interface. This allowed us to overcome a common limitation of many oil–water interfaces, concerning the minimum attainable interfacial tension (γ_max_ ≈ 32.5 mN/m for most edible oils at T = 21 °C) [42]. As shown earlier [24], in many of the mixed extract + pancreatin systems, γ_eq_ may drop below γ_max_ of the edible oil–water interface; hence, some of the experimental γ values could be biased at the oil–water interfaces. In the present study, we compared γ_eq_ for 1% of solutions of the nine saponin-rich extracts tested for their effect on pancreatin activity. The results collected in Table 4 show a wide range of values, with **Pwr** exhibiting the minimum and **St** showing the maximum equilibrium surface tension of the bare extracts. The surface activity of the latter extract is thus rather weak, lower than that of many other saponin-rich plant extracts [12]. On the other hand, the remaining extracts all reduced γ_eq_ below 43 mN/m, with **Pwr** and **Nr** (33.7 mN/m and 34.1 mN/m, respectively) performing comparably well to the low-molecular-weight synthetic surfactants like the anionic sodium dodecyl sulfate (SDS), cationic cetyltrimethylammonium chloride (CTAB), or nonionic ethoxylated alcohol surfactants [43,44]. These values are also close to those of the major bile salt component, sodium cholate (35.9 ± 0.3 mN/m) [24]. Except for the least surface-active **Nr**, all extracts reduced surface tension to levels below that of pancreatin (γ_eq_ = 50.9 mN/m for the saturated solution) [24].

In terms of the surface compression rheological parameters of the adsorbed layers formed on the surface of 1% extract solutions, **Ms** clearly stands out with its storage modulus, E’ = 188 mN/m (Table 4), which is one of the highest values reported for saponin-rich extracts [45,46] and clearly points to the ability of the extract components to form strongly interconnected interfacial structures, imposing a high surface elasticity. All other extracts showed moderate values of E’ in the range of 7.6 mN/m–32.5 mN/m, pointing to a rather weak structure formation within the adsorbed layers. In terms of surface viscous behavior, only two extracts showed the loss moduli, E”, in excess of 14 mN/m (**Ms** and **Hs**). For all other adsorbed layers, the liquid-like response was negligible. Overall, for almost all extracts and for the positive control (sodium cholate, E’ = 12.7 ± 0.4 mN/m, E” = 1.0 ± 0.3 mN/m), the surface compression rheological response of the adsorbed layers was predominantly elastic (E’ > E”). The only exception was **Hs**, for which a similar viscoelastic behavior (E’ ≈ E”) was already observed in previous studies [12,47].

For most of the mixed pancreatin-extract layers, surface elasticity increased with respect to the bare extract. Only for **Ms** was the mixed adsorbed layer less elastic than the bare extract (yet still showing a very high E’ > 100 mN/m). Interestingly, its surface viscosity modulus increased at the same time (E” > 50 mN). Among the remaining extracts, a notable increase in E’ and E” was observed for **Sh** and **Prr**. For the remaining mixed adsorbed layers, no distinct changes in E’ and E” were observed (Table 4), suggesting that their surface rheological properties were dominated by the extracts, not by pancreatin (for which E’ = 47.8 mN/m and E” = 10.9 mN/m). Overall, the most pronounced synergistic effect on surface activity was observed for **Prr**, where γ_eq_ dropped by ~9 mN/m, while E’ and E” increased by >10 mN/m.

The high surface activity of the saponin-rich extracts not only reduces their surface tension but may also influence the formation of oil-in-water emulsions and potentially modify the enzymatic lipid digestion process. On the one hand, the surface-active components of the extracts can stabilize emulsions in cases of bile acid deficiency. On the other hand, they could block enzyme access by occupying all available adsorption sites at the digested oil–water interface. The poor emulsification potential of the bare **Prr**, **Cr**, and **St** extracts correlates well with their moderate ability to reduce surface tension (γ_eq_ > 41 mN/m; see Table 4). However, the remaining extracts under the employed experimental conditions were even more efficient emulsifiers than the positive control, sodium cholate [24]. Nevertheless, when mixed with pancreatin, the emulsification potential of the extracts generally decreased (Figure 4d–f).

The molecular modeling results provide an additional explanation for most of the experimental observations. Given that extracts are multicomponent mixtures that complicate computational studies, we focused this work on saponins for their ability to modulate lipolytic activity through direct interactions with pancreatic lipase. Cholate, escin Ia, osladin, and ginsenoside Rg1 did not persistently occupy the catalytic pocket of pancreatic lipase during the simulations. Instead, these compounds were located outside or near the active-site region, particularly around the lid domain, β5 loop, and β9 loop. This binding pattern suggests that their effects are unlikely to result from direct blockage of the catalytic site. Rather, these compounds may modulate lipase activity indirectly by affecting lid opening, substrate accessibility, or the conformational dynamics of regions involved in interfacial activation. This interpretation is consistent with the strong lipase stimulation observed experimentally with sodium cholate and horse chestnut seed extract, which is rich in escins.

The results for the *Panax* extracts require a more careful interpretation. In experimental studies, ginseng extracts were among the strongest plant-derived stimulators of pancreatic lipolysis. However, the computational results showed that individual ginsenosides behaved differently. Ginsenoside Rg1 did not block the active site but increased fluctuations in the lid region and altered the distance between the catalytic triad residues during the simulation. Ginsenosides Re and Rd, and to a lesser extent Rb1, adopted active-site/β9-loop-associated poses and formed persistent contacts near residues that may influence substrate access. Therefore, the overall stimulatory effect of ginseng extracts likely reflects the combined effects of multiple ginsenosides and their interfacial interactions, rather than the action of a single compound. This may also explain why some extracts showed weak inhibition at low concentrations but stimulation at higher concentrations, where interfacial effects may become dominant.

The computational results also help explain why good emulsion stabilization does not always translate into strong lipase stimulation. *Herniaria glabra* extract stabilized emulsions well in the presence of pancreatin, but its lipase-stimulating effect was moderate compared with those of white ginseng and horse chestnut extracts. The modeled herniariasaponins interacted within or near the active-site region and contacted catalytic or nearby residues, which could partially counterbalance the favorable interfacial effect of the extract. Similarly, osladin did not block the active site in the simulations, yet the corresponding *Polypodium vulgare* extract produced only modest stimulation experimentally. This difference emphasizes that whole extracts contain mixtures of saponins, phenolics, and other constituents and that the behavior of a single representative compound cannot fully explain the biological response of the extract as a whole.

Taken together, the experimental and computational results support a proposed dual mechanism in which saponin-rich extracts influence lipolysis through both interfacial effects and compound-dependent interactions with pancreatic lipase. First, saponins can act as natural surfactants that modify the lipid–water interface, affect emulsion stability, and potentially improve substrate availability for pancreatic lipase. Second, individual saponins can interact with pancreatic lipase near the active site or regulatory structural regions, thereby altering local conformational dynamics and active-site accessibility. Stimulation is most likely observed when interfacial facilitation and non-blocking protein interactions occur together. Inhibition may occur when saponins occupy the active-site region, alter catalytic geometry unfavorably, or form highly elastic interfacial layers that limit enzyme access to the lipid substrate.

It should be emphasized that the computational models were constructed using selected individual saponins and the porcine pancreatic lipase–colipase complex in aqueous simulation conditions. They do not fully reproduce the complexity of pancreatin, the oil–water interface, or the complete chemical composition of the plant extracts. Therefore, the molecular simulations should be interpreted as mechanistic support for the experimental results rather than as direct quantitative predictors of extract activity.

Although certain saponins are associated with considerable toxicity, toxicity is not uniform across this chemically diverse group. Saponins occur naturally in commonly consumed foods, including soybeans, chickpeas, lentils, beans, and peanuts [48,49]. Their biological and toxicological effects depend strongly on their molecular structure and concentration. In particular, experimental structure–activity studies have demonstrated that the hemolytic activity of steroidal saponins depends on the aglycone structure and on the length, linkage, and substitution pattern of the sugar chains. The ProTox-3.0 analysis predicted lower acute oral toxicity for ginsenoside Rg1 than for sodium cholate, with predicted LD_50_ values of 4000 and 2000 mg kg^−1^, respectively [48]. Experimental evidence provides partial support for the predicted hepatotoxicity of sodium cholate. Hepatic injury and fibrosis have been reported in animal studies involving sodium cholate or cholic acid, with the observed effects depending on concentration, exposure duration, and experimental conditions [50,51]. However, these findings should be interpreted cautiously because the sodium cholate study combined the compound with a high-cholesterol diet, making it difficult to determine its individual contribution. Furthermore, evidence obtained for cholic acid, the parent acid of sodium cholate, cannot be attributed directly to its sodium salt. In vitro studies have also demonstrated concentration-dependent effects of sodium cholate, with cytotoxicity and apoptosis occurring primarily at supraphysiological concentrations, whereas lower concentrations were comparatively well tolerated [52]. Sodium cholate has also been reported to cause respiratory tract irritation following inhalation exposure [53]. However, such irritation does not directly confirm the broader respiratory-toxicity endpoint predicted by ProTox-3.0. Thus, although the predicted hepatotoxicity of sodium cholate has some experimental support, its predicted respiratory, cardiac, and immune toxicity endpoints should be considered preliminary computational alerts requiring targeted experimental confirmation. The toxicity predictions for the saponins investigated should also be interpreted cautiously. A complete ProTox-3.0 toxicity profile could be obtained only for one structurally defined saponin, ginsenoside Rg1, and the resulting prediction cannot be generalized to all saponins or to the chemically complex plant extracts investigated in this study. Saponin toxicity depends strongly on molecular structure, including the nature of the aglycone and the number and arrangement of sugar residues, as well as on concentration, exposure duration, and route of administration. Interestingly, although ProTox-3.0 identified a potential cardiotoxicity alert for ginsenoside Rg1, experimental studies have reported cardioprotective effects of this saponin in cellular and animal models [54,55,56]. This discrepancy further emphasizes that computational toxicity predictions should be interpreted as preliminary alerts rather than definitive evidence of toxicity.

Among herbal supplements, ginseng has been extensively studied in clinical trials, which have generally reported beneficial effects with relatively few adverse events [57]. Nonetheless, the present findings do not support the immediate or unrestricted replacement of bile salts with crude saponin-rich extracts. Rather, they indicate that selected, chemically standardized extracts or purified saponins may warrant further investigation as potential bile salt substitutes in controlled applications with carefully defined compositions and doses. Before practical implementation, their chemical composition should be characterized and their safety evaluated at the concentrations required to produce the desired lipase-stimulating and interfacial effects. Moreover, aquatic toxicity was not evaluated in the present study and will require dedicated experimental investigation before environmental or practical applications are considered, given the risk associated with some saponins acting as biopesticides [58]. Practical substitution of bile salts should be considered only after an adequate safety margin has been demonstrated between the functionally effective concentration and concentrations associated with toxicity.

## 4. Materials and Methods

### 4.1. Raw Materials and Preparation of Extracts

The plant materials used in the tests and the extract weights are shown in the Table 5. First, 10 g of dried raw material was extracted with 80 mL of a 70% ethanol solution in water under reflux for 10 days. The extracts were concentrated to dryness in a vacuum evaporator and then freeze-dried. The weight of plant extracts, English and Latin names, and abbreviations denoting extracts that are used in the manuscript are given below.

### 4.2. Reagents

*Pancreatin* was purchased from Sigma-Aldrich (Merck, Darmstadt, Germany; ingredients: pancreatic lipase, 40 PhEur units/mg; pancreatic α-amylase, 106 PhEur units/mg; pancreatic proteases, including trypsin, chymotrypsin, and elastase, 7.04 units/mg).

- *Tris* (hydroxymethyl)aminomethane—Roche, Mannheim, Germany;
- *HCl*, NaOH—Chempur, Piekary Śląskie, Poland;
- *Olive oil*—Monini Extra Virgin Olive Oil, exp. 10.07.2025, L1063183 11.36;
- *Ethanol* 95%, Polmos, Companies of the Spirytus Industry in Warsaw, Poland;
- *Thymolphthalein* Merck, Germany.

### 4.3. Apparatus

Analytical UHPLC separation was conducted on a Thermo Scientific UHPLC Ultimate 3000 apparatus (Thermo Fisher Scientific, Waltham, MA, USA) consisting of an LPG-3400RS quaternary pump with a vacuum degasser, a WPS-3000RS autosampler, and a TCC-3000SD column oven. The system was linked to an ESI-qTOF Compact HRMS detector (Bruker Daltonics, Bremen, Germany). Chromatographic analyses were performed on a Kinetex RP-18 column (100 mm × 2.1 mm × 2.6 µm; Phenomenex, Torrance, CA, USA) in a 50 min gradient mode. The solvent system consisted of 0.1% HCOOH in water (A) and acetonitrile (B) and was run according to the following elution program: 0 → 30 min (5 → 95% B), 30 → 40 min (95% B), 40 → 45 min (95 → 5% B), and 45 → 50 min (5% B). All UHPLC analyses were carried out isothermally at 30 °C. The injection volume was 2 µL, and the flow rate was 0.3 mL/min. The ESI-MS module was operated in the negative mode, and sodium formate cluster ions were used for mass calibration. The main instrumental parameters were as follows: scan range 200–2200 *m*/*z*; dry gas—nitrogen; temperature 200 °C; potential between the spray needle and the orifice: 4.2 kV. Collision energy in CID cells was 80 eV. For data collection and evaluation of the obtained mass spectra, the software version 5.3 (Bruker Daltonics, Bremen, Germany) was utilized.

- Lyophilizer (Christ Alpha 1-2 LO Plus, Osterode am Harz, Germany);
- BioTek Epoch Microplate Spectrophotometer Microplate Reader, 200–999 nm, Gen 5 (Agilent Technologies, Santa Clara, CA, USA);
- Laboratory water purification unit Integral 10 Milli-Q (Millipore, Burlington, VT, USA);
- Vacuum evaporator Buchi (Buchi, Rotavapor R-100, Flawil, Switzerland);
- Ultrasonic probe Sonopuls HD 2070 (Bandelin, Berlin, Germany);
- Profile Analysis Tensiometer PAT-1 (Sinterface Technologies, Berlin, Germany);
- Water bath with precise temperature control (AJL, LW 102, Kraków, Poland);
- pH meter (Oakton, WD-35419-03, Singapore).

### 4.4. Lipolytic Activity of Pancreatin

The lipolytic activity of pancreatin was measured according to the modified method described by Tietz and Fiereck [59]. First, 8 mL samples of extract solutions in 0.04 M Tris-HCl buffer, pH 8.0, with concentrations of 0, 0.04, 0.12, 0.35, 1.05, 3.1, and 9.4 mg/mL were added to Erlenmeyer flasks. In control samples without extract, 8 mL of 0.04 M Tris-HCl buffer (pH 8.0) was added to the Erlenmeyer flask. Then, 2 mL of olive oil was added to the sample, and the mixture was shaken vigorously to form an emulsion. Before adding the enzyme, samples were preincubated in a water bath at 37 °C for 10 min. To start the enzymatic reaction, 1 mL of pancreatin solution in 0.04M Tris-HCl buffer, pH 8.0, at 15 mg/mL was added to the sample. The samples were incubated for 3 h at 37 °C. Then, 3 mL of 95% ethanol in water was added to the samples to stop the enzymatic reaction. The amount of fatty acids released by lipase was measured by titrating the samples with 0.05 N NaOH against a 1% thymolphthalein solution in ethanol. The measurement was repeated 3 times, and the maximum error (ME) was calculated.

The rate of the reaction was expressed as the number of micromoles of fatty acids released by pancreatin per 1 mg of enzyme *C_FFA_* and was calculated according to Equation (1):

*C_FFA_* = (*V_Sml_*− *V_Cml_*) × 3.333 (1)

where *C_FFA_* is the number of micromoles of fatty acids hydrolyzed by pancreatin lipase per mg of pancreatin; *V_Sml_* is the number of milliliters of 0.05 N NaOH used for titration of the sample; and *V_Cml_* is the number of milliliters of 0.05 N NaOH for titration of the control sample.

The effect of the extract on pancreatic lipase activity at each extract concentration was expressed as a percentage (*A%*) and calculated using Formula (2).

(2) $$A\%=\frac{C_{FFA}}{C_{FFA0}}\cdot 100\%$$

where *A%* is the effect of the extract on lipase activity at each extract concentration expressed as a percentage; *C_FFA_* is the number of micromoles of fatty acids hydrolyzed by pancreatin at each extract concentration; and *C_FFA_*_0_ is the number of micromoles of fatty acids hydrolyzed in the sample without extract.

The effect of extracts on lipase activity was also presented as *A%_max_* and *Effect%. A%_max_* value was calculated in the same way as *A%* but for the maximum extract concentration (Equation (3)).

(3) $${A\%}_{max}=\frac{C_{FFAmax}}{C_{FFA0}}\cdot 100\%$$

where *A%_max_* is the relative activity of the sample compared to the control test (without extract), *C_FFAmax_* is the number of μmoles of released fatty acids per mg of pancreatin at the extract concentration 9.4 mg/mL, and *C_FFA_*_0_ is the number of μmoles of fatty acids released in the sample without extract.

*Effect%* is the difference between the lipase activity at the maximum concentration of extract and the sample without extract, in relation to the sample without extract, calculated according to Equation (4)

(4) $$Effect\%=\frac{C_{FFAmax}-C_{FFA0}}{C_{FFA0}}\cdot 100\%$$

where *Effect%* is the difference between the lipase activity at the maximum concentration of extract (9.4 mg/mL in reaction mixture, *C_FFAmax_*) and the sample without extract (*C_FFA_*_0_) in relation to the sample without extract. Positive *Effect%* values indicate stimulation of lipase activity; negative values indicate enzyme inhibition.

Relative error (RE) was calculated using the total differential method for the values *A%_max_* and *Effect%* with *n* = 3.

### 4.5. Colorimetric Measurement of Total Phenolic Compounds

Total phenolic compounds were measured using the modified method of Sari et al. [60]. First, 200 µL of the extract solution in 70% methanol, at a concentration of 20 mg/mL, was added to an Eppendorf tube. Then, 40 μL of Folin–Ciocalteu phenol reagent and 800 μL of 10% Na_2_CO_3_ aqueous solution were added to the sample.

Samples were incubated for 30 min at room temperature and centrifuged (12600 RCF). Then, 50 µL of each sample was added to the wells of a microwell plate. The absorbance was measured at 725 nm using a microplate reader. In blank samples, methanol was used instead of the extract solution. The total phenol content was expressed as gallic acid equivalents (GAEs), determined from a calibration curve. Each measurement was repeated three times, and the standard deviation (SD) was calculated.

### 4.6. Measurement of the Amount of Flavonoids Using the Colorimetric Method

The total amount of flavonoids was measured using the method of Sari et al. [60]. First, 50 µL of the extract solution in 70% methanol at 20 mg/mL and 50 µL of 2% AlCl_3_ in methanol were added to the wells of a microwell plate. Samples were stored in the dark at room temperature for 60 min. The absorbance was measured using a microplate reader at λ = 420 nm. A blank sample was also prepared using 70% methanol instead of the extract solution. The flavonoid content was presented as quercetin equivalents (QEs). Each measurement was performed in triplicate, and the standard deviation (SD) was calculated.

### 4.7. Measurement of the Ability of Extracts to Scavenge the Abts^•+^ Radical Cation

The antiradical activity of extracts was measured by the method described by Le Grandois et al. using the ABTS^•+^ cation radical [61].

A 7 mmol/L ABTS solution in water and a 2.45 mmol/L sodium persulfate solution in water were prepared. The two solutions were mixed in a 1:1 ratio and then stored in the dark for 16 h to generate the ABTS^•+^ cation radical. The measurement was performed as follows: 200 μL of the ABTS^•+^ radical solution and 2 μL of extract solution in 70% MeOH at 6.67 mg/mL were added to wells in a microwell plate. The absorbance was measured 15 min later at a wavelength λ = 734 nm. The measurement was performed three times, and the maximal error (ME) was calculated. The antiradical activity was expressed as Trolox equivalents (Trolox [mM]).

### 4.8. Antioxidant Activity of Extracts Measured with the Frap (Ferric Reducing Antioxidant Power) Method

The analysis was performed using the method of Jimenez-Alvarez et al. [62].

First, the FRAP reagent was prepared as follows: 78 mg of TPTZ (2,4,6-tris(2-pyridyl)-s-triazine) and 135 mg of FeCl_3_ × 6H_2_O were dissolved in 25 mL of 0.3 M acetate buffer, pH 3.6 (CH_3_COOH: CH_3_COONa).

The measurement was performed as follows: 20 µL of extract solution at 6.7 mg/mL in 70% methanol and 200 µL of FRAP reagent were added to the wells of a microwell plate. Absorption was measured after 4 min at λ = 593 nm.

The results were expressed as mg Fe^2+^ per mL of reaction mixture (Fe^2+^ [mg/mL]).

### 4.9. Measurement of Equilibrium Surface Tension, Surface Elasticity Modulus, and Surface Viscosity Modulus

Milli-Q water was used to prepare all solutions for surface tension and rheology measurements. The extract powders alone or mixed with pancreatin were dissolved in Milli-Q water (1% each) and filtered through a 5 μm syringe filter immediately prior to the surface tension/surface rheology measurement using a drop profile analysis tensiometer PAT-1. Temperature was maintained at 21 °C with a thermostatic bath. A drop of the tested extract (or extract + pancreatin) solution (10 µL) was formed at the tip of a steel capillary immersed in a glass cuvette (20 mL) filled with air. All experiments were performed at least four times. In the first part of the measurement (0–600 s), the drop volume was kept constant (10 µL), providing information about the dynamic surface tension (i.e., surface tension vs. time). The equilibrium surface tension (γ_eq_) was calculated by extrapolating the dynamic surface tension to infinite time [63]. In the second part of each measurement, the drop area was subjected to sinusoidal oscillations with a relative amplitude of 4% and a frequency of 0.1 Hz. The analysis of the surface tension response to the drop area oscillations provided the elastic (E’) and viscous (E”) parts of the complex surface compression viscoelastic modulus (surface elasticity modulus and surface viscosity modulus) [64].

### 4.10. Emulsification Tests

First, 1 mL of 1% extract solution of each plant extract or sodium cholate was homogenized with 0.2 mL of olive oil stained with Sudan Red IV (0.02%) using an ultrasonic probe (Bandelin (Berlin, Germany) Sonopuls HD 2070; 30 s, 20% cycle, 30% max. power). Emulsions were stored at room temperature and photographed at the beginning and after 1 h and 24 h.

### 4.11. UHPLC-ESI-MS and MS/MS Analyses

For UHPLC-ESI-MS analyses, the extracts were dissolved in 70% methanol (1 mg of extract per 1 mL), filtered through a 0.22 µm PTFE syringe filter (Merck-Millipore, Darmstadt, Germany), and stored at room temperature before analysis. Analytical UHPLC separation was conducted according to our previous publication [24].

### 4.12. Statistical Analysis

The relative error (RE) and maximum error (ME) were calculated using the total differential method. The nonparametric statistical test (Kruskal–Wallis test of ranks, one-way ANOVA on ranks) was performed to assess the statistical significance of differences between the samples. Post hoc tests were performed to determine which groups differed significantly from one another. Dunn’s test was performed with the Benjamini–Hochberg correction for multiple comparisons. The groups that did not differ significantly are marked with the same letters (a, b, c, etc.); the lack of a letter above the bar in figures means that the value differs significantly from all others.

### 4.13. Computational Details

To further examine the differences in saponin activities, their mechanisms of interaction with porcine lipase–colipase were investigated. The crystal structure of the porcine pancreatic lipase–colipase complex (PDB ID: 1ETH) was used as the starting model for all calculations. It comprised lipase, colipase, tetraethylene glycol monooctyl ether (TGME) bound to the active site of the lipase, and two oligosaccharide molecules (beta-D-mannopyranose-(1-3)-[beta-D-mannopyranose-(1-6)]beta-D-mannopyranose-(1-4)-2-acetamido-2-deoxy-beta-D-glucopyranose-(1-4)-2-acetamido-2-deoxy-beta-D-glucopyranose) at the glycosylation site. The Schrödinger software version 2026-1 package was used for all calculations. Repeated chains (C and D) and the co-crystallized ligand were deleted, as well as all water molecules and all entries associated with the protein chains C and D. Protein structure was prepared using Schrödinger’s Protein Preparation Wizard. This procedure included assigning bond orders, replacing hydrogen atoms, and optimizing the hydrogen-bonding network using the OPLS4 force field [65]. Protonation states of ionizable residues were assigned using Epik (https://www.schrodinger.com/platform/products/epik/, (accessed on 19 July 2026)) [66] at pH 7.4 to reflect physiological conditions, and the structure was subjected to restrained energy minimization to remove unfavorable contacts while maintaining the overall protein geometry.

Ligand structures, including cholate, ginsenoside Rg1, ginsenoside Re, ginsenoside Rb1, ginsenoside Rd, herniariasaponin 2, herniariasaponin 4, osladin, hederacoside C, and escin Ia, were retrieved from PubChem (https://pubchem.ncbi.nlm.nih.gov/, (accessed on 19 July 2026)) and prepared using LigPrep (https://www.schrodinger.com/platform/products/ligprep/, (accessed on 19 July 2026)), where three-dimensional conformations were generated, and protonation states were assigned using Epik at pH 7.4. Stereochemical configurations were preserved, and the resulting structures were energy minimized to obtain suitable starting geometries for docking. Receptor grids were generated using Glide (https://www.schrodinger.com/platform/products/glide/, (accessed on 19 July 2026)) to represent multiple potential ligand-binding regions on the lipase surface. In our previous work [24], we investigated two glycan-binding sites for saponin binding and found that some saponins were also capable of binding within the active-site region. Therefore, in the present study, three distinct binding sites were used for docking studies. For each site, the inner grid box was set to 10 Å, while the outer box dimensions were 36 Å. Docking calculations were performed using Glide in Extra Precision (XP) mode [67]. An iterative docking strategy was applied in three stages: initial docking runs identified candidate binding modes in the first binding site, followed by additional grid generation and docking rounds with refined or expanded grids. Final poses were selected based on Glide XP scores and consistency of interactions with surrounding residues. Selected docking complexes were further evaluated using Prime MM-GBSA calculations with the OPLS4 force field and the VSGB2.1 implicit solvent model [68], considering a flexible region extending 20 Å from the ligand. MM-GBSA calculations were carried out in two scenarios: binding of the ligand to the empty receptor and to the receptor in the presence of two other ligands.

Molecular dynamics simulations were carried out using Desmond [69] to assess the stability of selected ligand–protein complexes and changes in the enzyme’s structure, which could cause the modulation of its affinity. Systems were solvated in an orthorhombic box (absolute size 100 Å) using the TIP3P water model and neutralized with 0.15 M NaCl. A multistep relaxation protocol was applied prior to production runs, including initial low-temperature dynamics followed by gradual heating and equilibration under both NVT and NPT conditions. Production simulations were performed in the NPT ensemble at 300 K and 1 atm using the Nose–Hoover thermostat and Martyna–Tobias–Klein barostat. Simulations were run for 500 ns, and trajectory frames were recorded at regular intervals for analysis. Trajectories were subsequently analyzed using the Simulation Interaction Diagram and the Schrödinger Python module (https://www.schrodinger.com/python-api/, (accessed on 19 July 2026)) to evaluate protein stability, ligand-binding behavior, and the persistence of key intermolecular interactions over the simulation period. Specific measurements to describe the extent of the lid and the distance between the residues were made using the protocol described in [24].

Toxicity assessment was carried out using the ProTox 3.0 predictive web server (https://tox.charite.de/protox3/, (accessed on 19 July 2026)) [70]. The PubChem names of sodium cholate and ginsenoside Rg1 were used as inputs. For all other saponins, ProTox 3.0 predictions were not possible due to size limitations.

## 5. Conclusions

This study demonstrated that selected saponin-rich plant extracts can modulate pancreatin’s lipolytic activity. The strongest stimulation of pancreatic lipase was observed with sodium cholate, a bile salt reference. At the same time, the most active plant-derived extracts were obtained from white ginseng root and horse chestnut seeds. Other *Panax* extracts also stimulated lipolysis, supporting the importance of dammarane-type triterpene saponins in this effect. In contrast, *Aesculus* × *marylandica* seed peel extract inhibited lipase activity, indicating that strong surface activity and emulsion stabilization do not necessarily enhance enzymatic lipid digestion.

The experimental results indicate that the effect of saponin-rich extracts on lipolysis depends on multiple mechanisms. Molecular docking, MM-GBSA calculations, and 500 ns molecular dynamics simulations showed that representative saponins differ substantially in their binding preferences and effects on pancreatic lipase dynamics. Hederacoside C, herniariasaponins 2 and 4, and ginsenosides Re, Rd, and Rb1 illustrated inhibitor-like binding behavior, interacting with the catalytic triad or nearby active-site residues, or blocking the active site sterically. Cholate, escin Ia, osladin, and ginsenoside Rg1 did not persistently block the catalytic pocket and showed more activator-like behavior. The simulations also showed ligand-dependent changes in the lid, β9 loop, and catalytic-site geometry, supporting the idea that saponins may modulate lipase activity by altering conformational dynamics and active-site accessibility. Because the computational work was performed on selected individual saponins rather than on complete extract mixtures, future studies should examine purified compounds, defined mixtures, and models that include the lipid–water interface to better link individual constituents to extract-level biological activity.

The antioxidant part of the study showed a strong positive correlation between total phenolic content and antioxidant/antiradical activity, whereas flavonoid content did not correlate as strongly with these activities. The highest amount of phenolic compounds was noted in the extracts from sycamore twigs, while the lowest amount of phenols was found in the extract from American ginseng root. The highest amount of flavonoids was found in the extract from the herb smooth rupturewort; the lowest amount was found in the extract from the root of American ginseng.

Thus, the extracts combine two potentially valuable properties: antioxidant activity, associated mainly with phenolic compounds, and modulation of lipid digestion, associated mainly with saponins and their interfacial behavior. However, these findings should be regarded as a basis for further mechanistic and safety studies rather than as support for immediate therapeutic application; although ginsenoside Rg1 showed lower predicted acute oral toxicity than sodium cholate, the safety of purified saponins and chemically standardized extracts must be confirmed experimentally at functionally effective concentrations before practical use.

## Figures and Tables

**Table 1:** *A%max* and *Effect%* values showing stimulation or inhibition of pancreatic lipase by extracts. Negative *Effect%* values indicate inhibition of lipase activity (**Ms** extract).

Extract ^1^	*A%max* ± *RE* ^2^	*Effect%* ± *RE* ^2^
**St**	160 ± 10	60.2 ± 25.9
**Ms**	31.7 ± 18.0	−68.3 ± 16.3
**Pwr**	517 ± 17	417 ± 21
**Prr**	360 ± 9	260 ± 13
**Ar**	431 ± 8	331 ± 10
**Nr**	350 ± 17	250 ± 24
**Sh**	294 ± 16	194 ± 25
**Hs**	459 ± 18	359 ± 23
**Cr**	189 ± 15	89 ± 31
**Ca**	801 ± 10	701 ± 11

^1^ Extract names: *Acer pseudoplatanus* (twigs)—**St**, *Aesculus marylandica* (seed peels)—**Ms**, *Panax ginseng* (white root)—**Pwr**, *Panax ginseng* (red root)—**Prr**, *Panax quinquefolius* (root)—**Ar**, *Panax notoginseng* (root)—**Nr**, *Herniaria glabra* (herb)—**Sh**, *Aesculus hippocastanum* (seeds)—**Hs**, *Polypodium vulgare* (rhizome)—**Cr**, sodium cholate **Ca**. ^2^ RE relative error (*n* = 3).

**Table 2:** Antiradical (Trolox) and antioxidant (Fe^2+^) activities and total amounts of phenols (GAE) and flavonoids (QE).

Extract ^1^	Trolox [mM] ± SD ^2^	Fe^2+^ [mg/mL] ± SD ^2^	GAE ± SD ^2^	QE ± SD ^2^
**St**	1.92 ± 0.02	7.20 ± 0.12	133.3 ± 8.1	80.24 ± 2.54
**Ms**	1.48 ± 0.07	1.47 ± 0.06	73.9 ± 3.6	19.74 ± 0.89
**Pwr**	0.42 ± 0.03	0.33 ± 0.02	15.8 ± 2.2	5.54 ± 1.07
**Prr**	0.65 ± 0.06	0.42 ± 0.02	32.3 ± 4.1	8.40 ± 1.08
**Ar**	0.33 ± 0.05	0.24 ± 0.01	11.7 ± 0.6	2.35 ± 0.98
**Nr**	0.29 ± 0.04	0.25 ± 0.03	12.2 ± 2.2	12.62 ± 1.39
**Sh**	1.75 ± 0.01	2.17 ± 0.06	105.3 ± 4.7	284.70 ± 17.40
**Hs**	0.94 ± 0.05	0.78 ± 0.08	57.4 ± 7.3	102.13 ± 10.28
**Cr**	1.90 ± 0.01	3.85 ± 0.13	121.7 ± 21.2	57.41 ± 2.12

^1^ Extract names: *Acer pseudoplatanus* (twigs)—**St**, *Aesculus marylandica* (seed peels)—**Ms**, *Panax ginseng* (white root)—**Pwr**, *Panax ginseng* (red root)—**Prr**, *Panax quinquefolius* (root)—**Ar**, *Panax notoginseng* (root)—**Nr**, *Herniaria glabra* (herb)—**Sh**, *Aesculus hippocastanum* (seeds)—**Hs**, *Polypodium vulgare* (rhizome)—**Cr**. ^2^ SD—standard deviation (*n* = 3).

**Table 3:** Putative identification of the most prominent compounds in our untargeted LC-MS analysis of the extracts from the investigated *Panax* species.

Rt (min)	Putative Identification	Measured *m*/*z* [M + HCOO]^−^	Theoretical *m*/*z* [M + HCOO]^−^	Molecular Formula (Neutral)	Error (∆ ppm)	MS/MS Fragments	Pwr	Ar	Prr	Nr
11.05	20-glucoginsenoside Rf or isomer	1007.5444	1007.5432 (961)	C_48_H_82_O_19_	1.16	961, 799, 637, 553, 475, 391	+	−	−	+
11.31	notoginsenoside FP1 or isomer	977.5326	977.5327	C_47_H_80_O_18_	0.10	769, 637, 475, 391	+	−	−	+
11.74	ginsenoside Rg1 * or isomer	845.4914	845.4904	C_42_H_72_O_14_	1.18	796, 637, 475, 391	+	+	+	+
11.80	ginsenoside Re or isomer	991.5482	991.5483	C_48_H_82_O_18_	0.10	945, 783, 637, 475, 391	+	+	+	+
13.88	pseudoginsenoside F11 or isomer	845.4922	845.4904	C_42_H_72_O_14_	2.12	799, 653, 491, 415	−	+	−	−
14.22	ginsenoside Rf or isomer	845.4917	845.4904	C_42_H_72_O_14_	1.54	799, 637, 475, 391	+	−	+	−
14.32	ginsenoside Ra3 or isomer	1285.6438	1285.6434 (1239)	C_59_H_100_O_27_	0.31	1239, 1107, 945, 783, 621, 353	−	−	−	+
14.67	notoginsenoside R2 or isomer	815.4824	815.4798	C_41_H_70_O_13_	3.2	637, 475, 391	+	−	+	+
14.87	notoginsenoside-FZ or isomer	1255.6324	1255.6328	C_58_H_98_O_26_	0.3	1209, 1077, 945, 915, 783, 621, 425/459	+	−	+	−
14.99	ginsenoside Rb1 or isomer	1153.6000	1153.6011	C_54_H_92_O_23_	1.0	1107, 945, 783, 621, 459	+	+	+	+

* Extracts: *Panax ginseng* (white root)—**Pwr**, *Panax ginseng* (red root)—**Prr**, *Panax quinquefolius* (root)—**Ar**, *Panax notoginseng* (root)—**Nr**. + present, − absent.

**Table 4:** Equilibrium surface tension (γ_eq_), surface elasticity modulus (E’), and surface viscosity modulus (E”) of the extracts (1% solutions).

Extract ^1^	γ_eq_ [mN/m] ± SD ^2^		E’ [mN/m] ± SD ^2^		E” [mN/m] ± SD ^2^	
	Bare Extract (1%)	Extract (1%) + Pancreatin (1%)	Bare Extract (1%)	Extract (1%) + Pancreatin (1%)	Bare Extract (1%)	Extract (1%) + Pancreatin (1%)
**Hs**	40.3 ± 0.4	41.7 ± 0.6	32.5 ± 1.8	11.6 ± 1.2	27.9 ± 1.3	6.1 ± 2.4
**Pwr**	33.7 ± 0.2	38.3 ± 0.3	15.2 ± 1.4	12.5 ± 0.9	4.2 ± 0.4	2.7 ± 0.4
**Ms**	39.1 ± 0.3	33.7 ± 0.1	188.3 ± 6.6	117.8 ± 9.9	14.5 ± 2.7	50.1 ± 7.9
**Sh**	37.8 ± 0.2	33.3 ± 0.8	7.8 ± 0.5	27.8 ± 3.4	3.1 ± 0.0	9.8 ± 1.5
**Ar**	38.8 ± 0.1	39.4 ± 1.1	10.0 ± 0.4	12.9 ± 3.0	2.4 ± 0.8	3.6 ± 1.6
**Prr**	41.2 ± 0.1	32.6 ± 0.3	9.3 ± 0.5	28.9 ± 1.9	1.7 ± 0.4	16.8 ± 0.4
**Nr**	34.1 ± 0.4	39.4 ± 1.6	18.4 ± 1.2	12.2 ± 4.0	4.0 ± 1.3	2.3 ± 1.0
**Cr**	42.6 ± 0.9	51.5 ± 0.2	11.1 ± 1.0	2.5 ± 0.7	2.3 ± 1.0	0.6 ± 0.0
**St**	58.4 ± 0.6	55.4 ± 0.6	7.6 ± 1.4	9.6 ± 0.4	2.5 ± 1.3	2.8 ± 0.4

^1^ Extracts: *Acer pseudoplatanus* (twigs)—**St**, *Aesculus marylandica* (seed peels)—**Ms**, *Panax ginseng* (white root)—**Pwr**, *Panax ginseng* (red root)—**Prr**, *Panax quinquefolius* (root)—**Ar**, *Panax notoginseng* (root)—**Nr**, *Herniaria glabra* (herb)—**Sh**, *Aesculus hippocastanum* (seeds)—**Hs**, *Polypodium vulgare* (rhizome)—**Cr**, *n* = 3, SD standard deviation. ^2^ SD—standard deviation (*n* = 3).

**Table 5:** Extracts used in this study.

Botanical Name of the Species	Common Name	Plant Part (*Latin*)	Abbreviation for the Extract	Weight of Extract [g]
*Aesculus hippocastanum*	Horse chestnut	Seeds (*semen*)	**Hs**	2.815
*Aesculus marylandica*	Maryland chestnut	Seeds’ peels (*semen shells*)	**Ms**	1.736
*Panax ginseng* (white)	Ginseng (white)	Root (*radix*)	**Pwr**	2.224
*Panax ginseng* (red)	Ginseng (root)	Root (*radix*)	**Prr**	3.111
*Panax notoginseng*	Notoginseng	Root (*radix*)	**Nr**	2.057
*Panax quinquefolius*	American ginseng	Root (*radix*)	**Ar**	3.266
*Herniaria glabra*	Smooth rupturewort	Herb (*herba*)	**Sh**	1.277
*Polypodium vulgare*	Common polypody	Rhizome (*rhizoma*)	**Cr**	1.967
*Acer pseudoplatanus*	Sycamore maple	Twigs (*viminibus*)	**St**	2.154

## Data Availability

Original contributions presented in this study are included in the text. Further inquiries can be directed to the corresponding author.

## Supplementary Materials

The following supporting information can be downloaded at https://www.mdpi.com/article/10.3390/molecules31152600/s1, Figure S1: Protein root mean square deviation (RMSD) values throughout a 500 ns molecular dynamics simulation for saponin–lipase–colipase complexes; Figure S2: Protein root mean square fluctuation (RMSF) values throughout a 500 ns molecular dynamics simulation for saponin–lipase–colipase complexes; Figure S3: Ligand–protein contacts were formed throughout the entire 500 ns molecular dynamics simulation and during the last 50 ns; Figure S4: Ligand root mean square deviation (RMSD) values throughout a 500 ns molecular dynamics simulation for saponin–lipase–colipase complexes; Figure S5: Ligand root mean square fluctuation (RMSF) values throughout a 500 ns molecular dynamics simulation for saponin–lipase–colipase complexes.

## Abbreviations

PDB ID	Protein Data Bank identifier
TGME	Tetraethylene glycol monooctyl ether
XP	Extra Precision docking mode
MM-GBSA	Molecular mechanics generalized Born surface area
MD	Molecular dynamics
RMSD	Root mean square deviation
RMSF	Root mean square fluctuation
OPLS4	Optimized Potentials for Liquid Simulations 4 force field
VSGB2.1	Variable-Dielectric Surface Generalized Born 2.1 implicit solvent model
TIP3P	Transferable Intermolecular Potential with 3 Points water model
NVT	Constant number of particles, volume, and temperature ensemble
NPT	Constant number of particles, pressure, and temperature ensemble
