# Supplementary material for: Interfacial and Molecular Mechanisms of Pancreatic Lipase Modulation by Saponin-Rich Extracts with Antioxidant Activity
Source: Molecules. 2026 Jul 25;31(15):2600. doi: 10.3390/molecules31152600 (PMC13468043; doi:10.3390/molecules31152600)
Supplement: Supplementary file 1 [file molecules-31-02600-s001.zip › molecules-4416101-supplementary.pdf]

molecules

MDPI

Supporting Information

Lipolytic Stimulation and Antioxidant Activity of Saponin-Rich Extracts: Experimental and In Silico Study

Zbigniew Sroka[1,*], Karina Kapusta[2,3], Senal D. Liyanage[3], Ta'Miyia Tobias[3], Victoria Petosyan[3,4], Wojciech Kolodziejczyk[2], Karolina Imiełowska[5], Michał Gleńsk[1], Beata Żbikowska[1], Andrzej Gamian[6], and Kamil Wojciechowski[7]

Figure S1. Protein Root Mean Square Deviation (RMSD) values throughout a 500 ns molecular dynamics simulation for saponin-lipase-colipase complexes.

Ligand	RMSD
**Cholate**	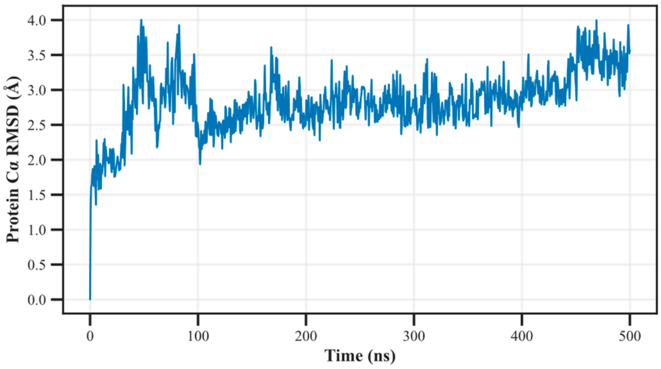
**Escin Ia**	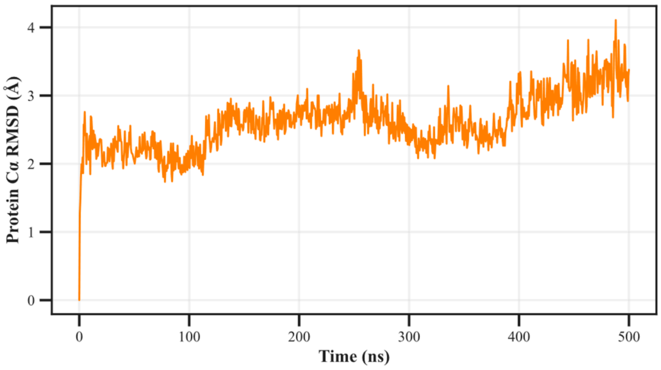
**Hederacoside**	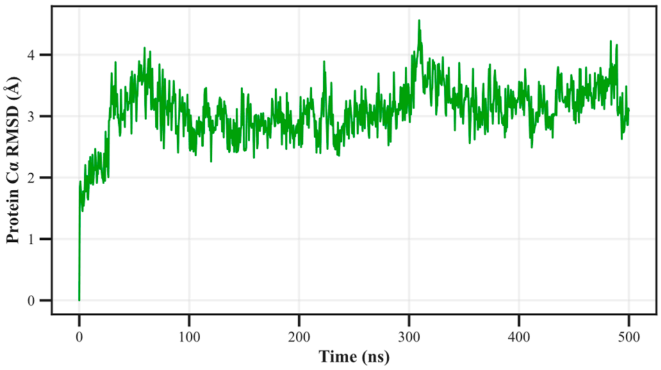

<table>
<tr><td>Ginsenoside Rb1</td><td>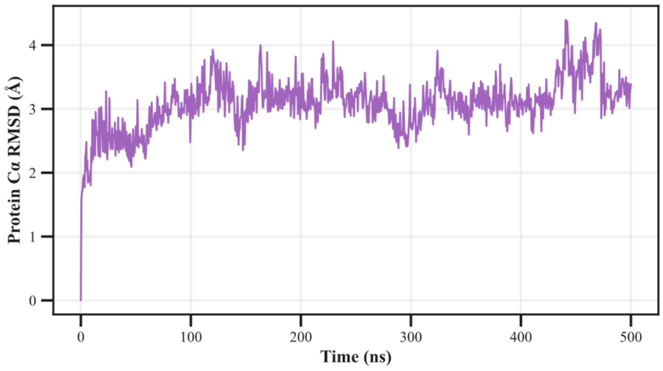
</td></tr>
<tr><td>Ginsenoside Re</td><td>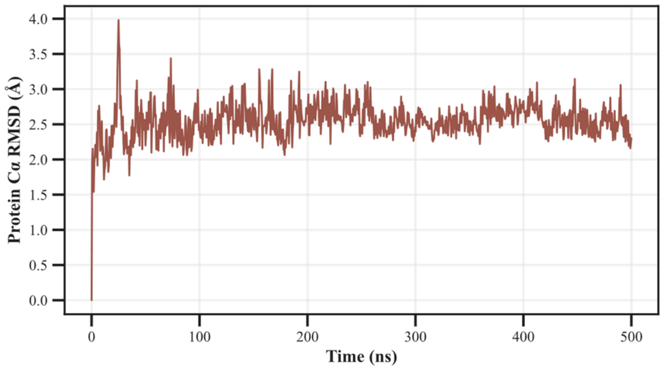
</td></tr>
<tr><td>Ginsenoside Rd</td><td>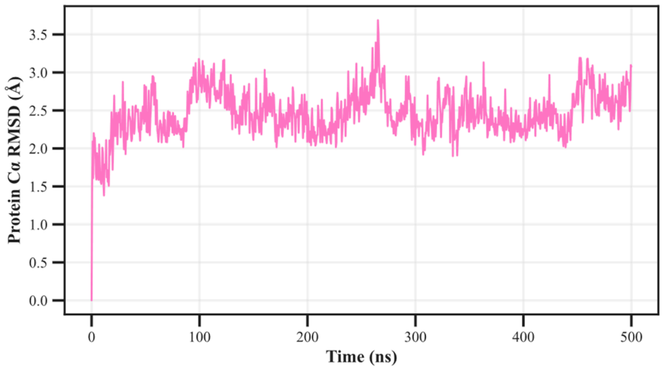
</td></tr>
<tr><td>Ginsenoside Rg1</td><td>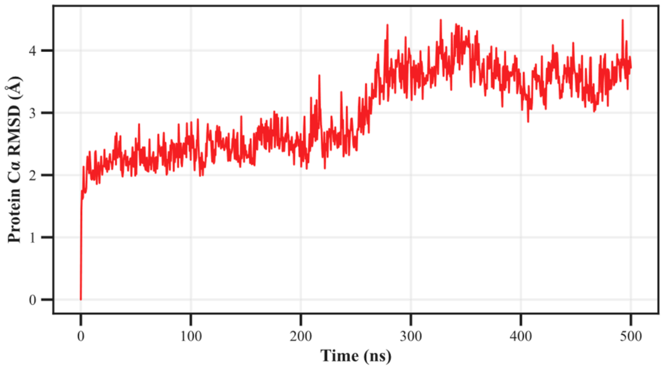
</td></tr>
</table>

**Osladin**	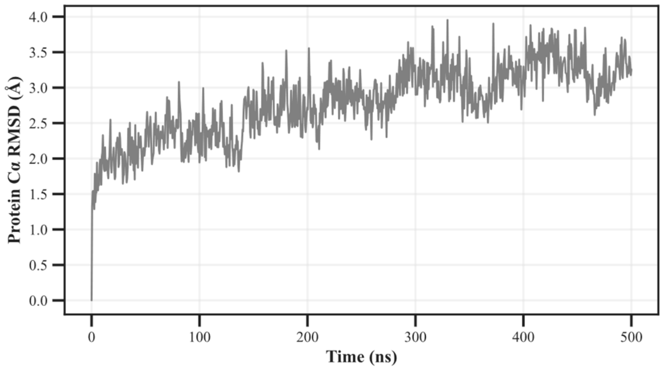
**Herniariasaponin 2**	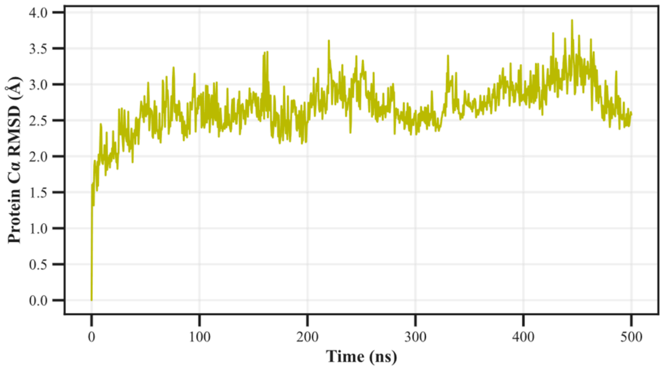
**Herniariasaponin 4**	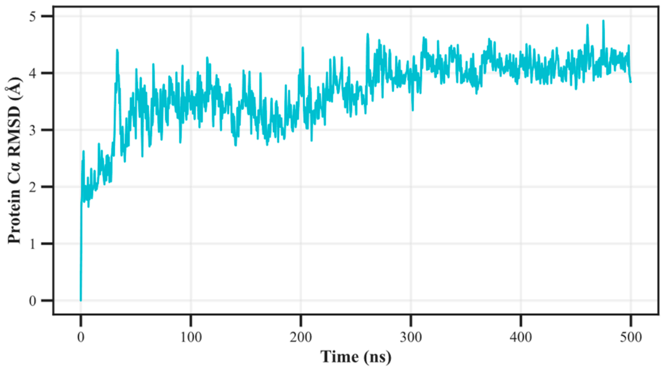

Figure S2. Protein Root Mean Square Fluctuation (RMSF) values throughout a 500 ns molecular dynamics simulation for saponin-lipase-colipase complexes.

<table>
<tr><th>Ligand</th><th>RMSF</th></tr>
<tr><td>Cholate</td><td>
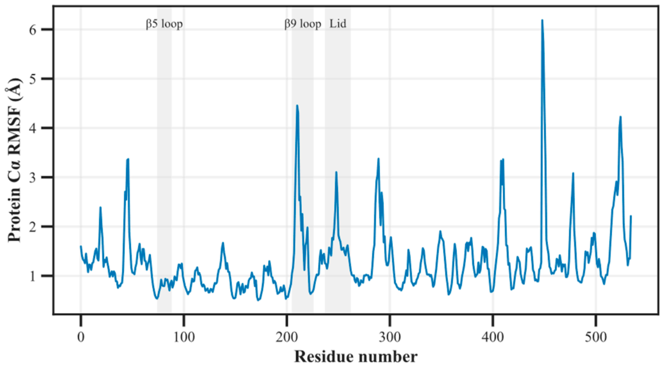

</td></tr>
<tr><td>Escin Ia</td><td>
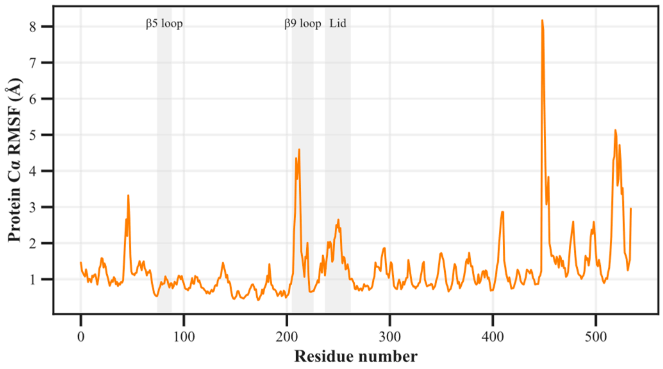

</td></tr>
<tr><td>Hederacoside</td><td>
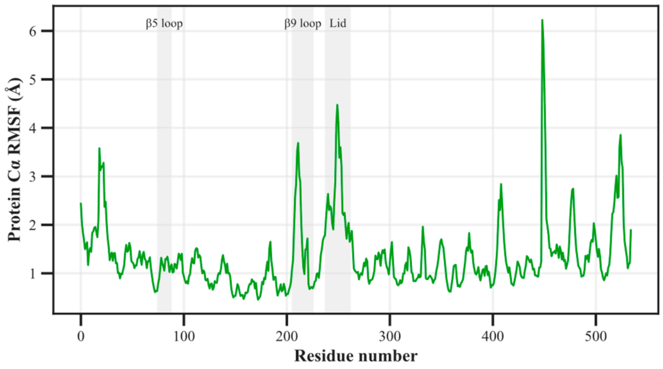

</td></tr>
<tr><td>Ginsenoside Rb1</td><td>
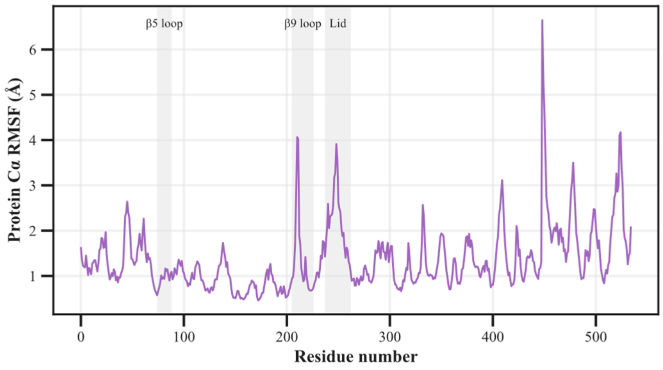

</td></tr>
</table>

**Ginsenoside Re**	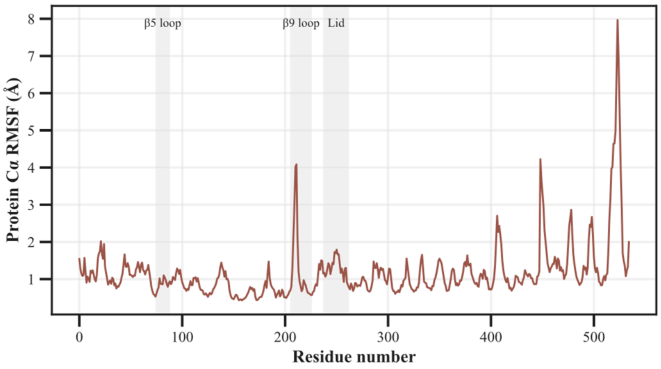
**Ginsenoside Rd**	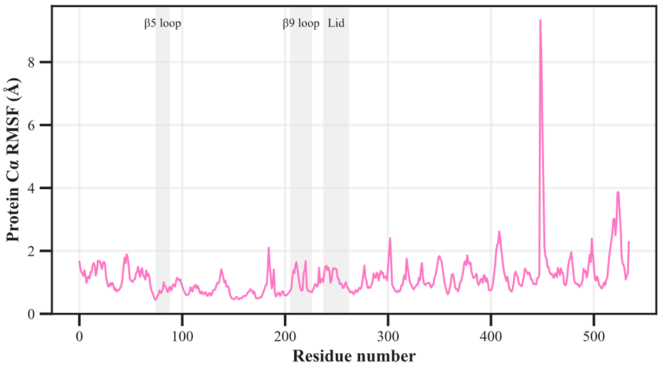
**Ginsenoside Rg1**	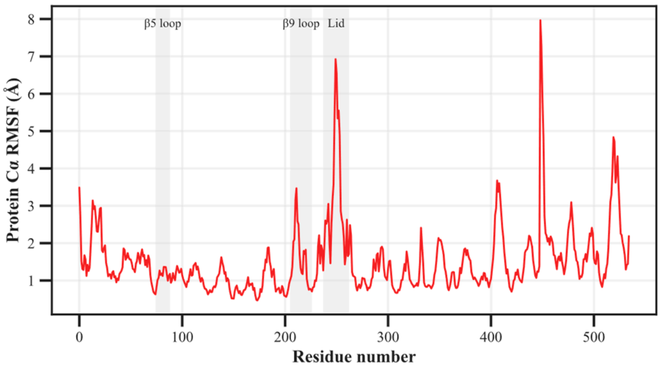
**Osladin**	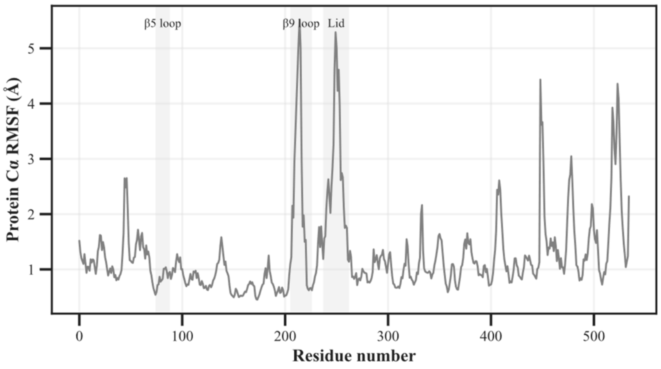

<table>
<tr><td>Herniariasaponin 2</td><td>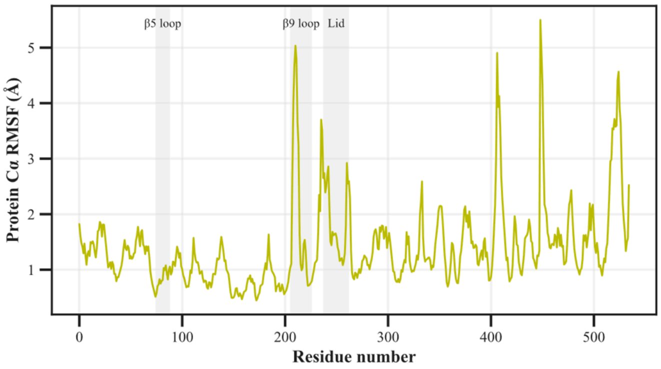
</td></tr>
<tr><td>Herniariasaponin 4</td><td>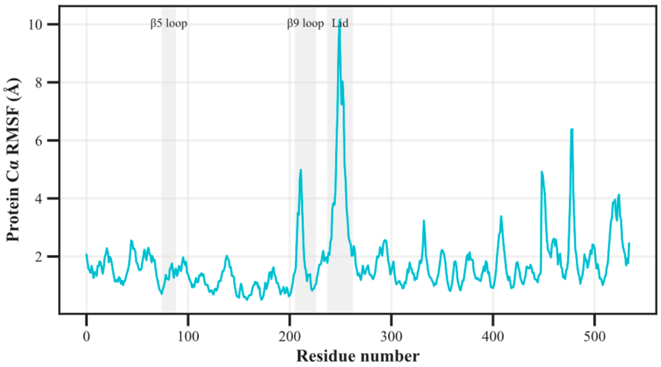
</td></tr>
</table>

Figure S3. Ligand-protein contacts were formed throughout the entire 500 ns molecular dynamics simulation and during the last 50 ns.

<table>
<tr><th>Ligand</th><th>Contacts (Full Trajectory)</th><th>Contacts (Last 50 ns)</th></tr>
<tr><td>Cholate
(Site 1)</td><td>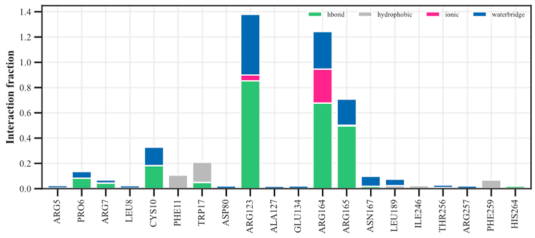
</td><td>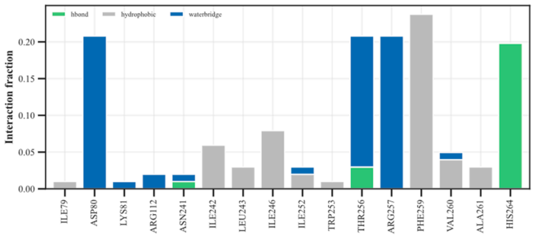
</td></tr>
<tr><td>Cholate
(Site 2)</td><td>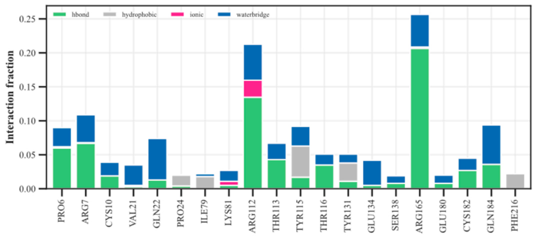
</td><td>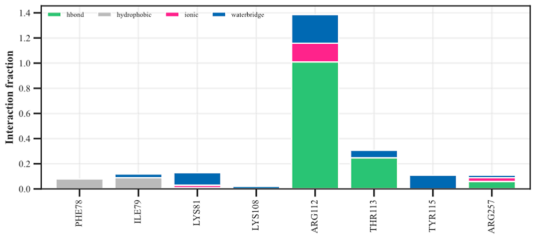
</td></tr>
<tr><td>Cholate
(Site 3)</td><td>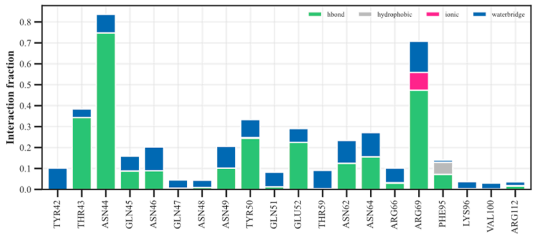
</td><td>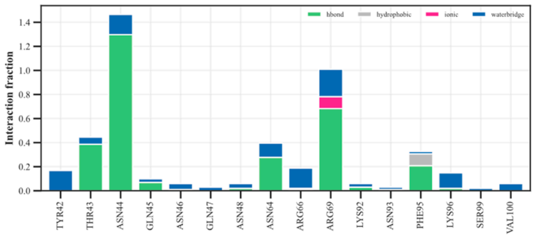
</td></tr>
</table>

**Escin Ia (Site 1)**	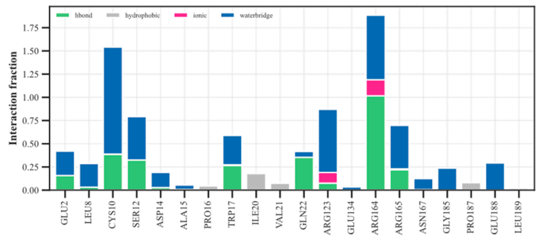	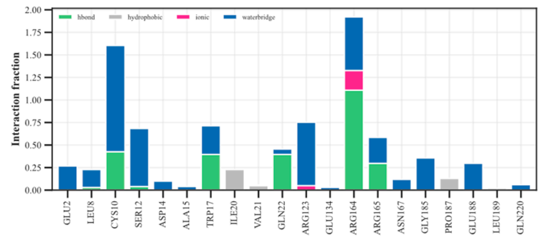
**Escin Ia (Site 2)**	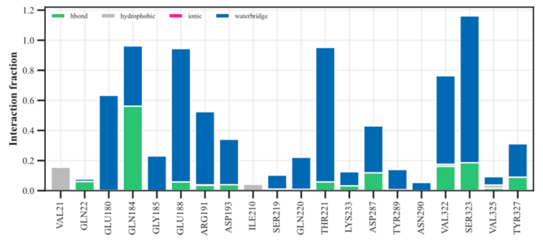	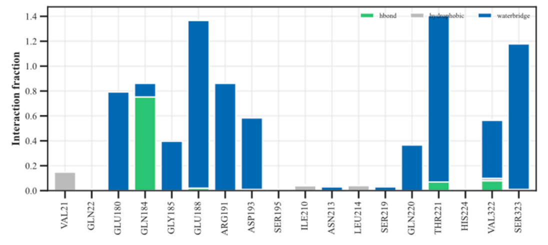
**Escin Ia (Site 3)**	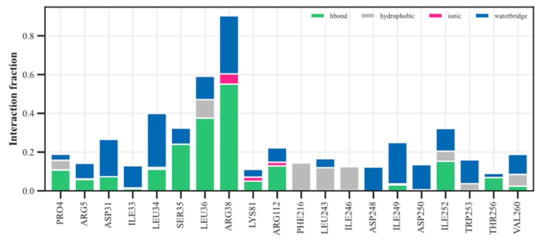	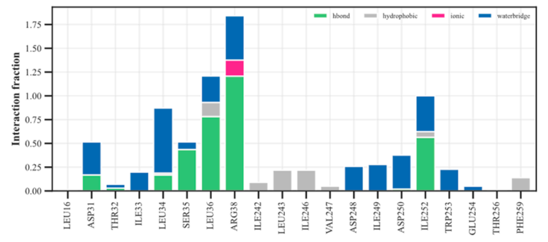
**Hederacoside C (Site 1)**	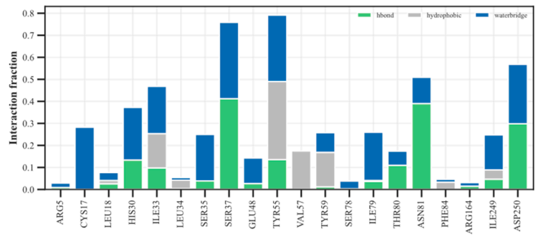	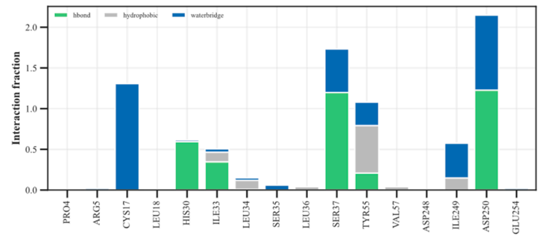
**Hederacoside C (Site 2)**	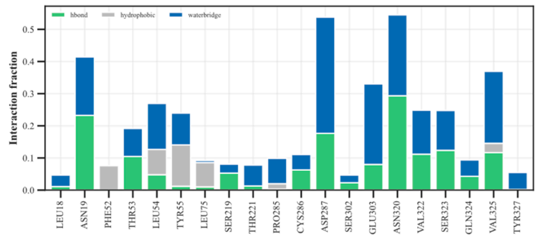	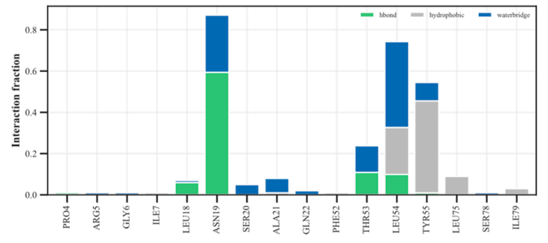
**Hederacoside C (Site 3)**	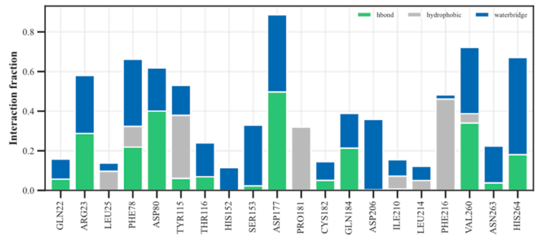	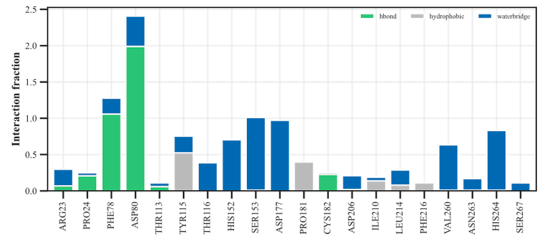
**Ginsenoside Rb1 (Site 1)**	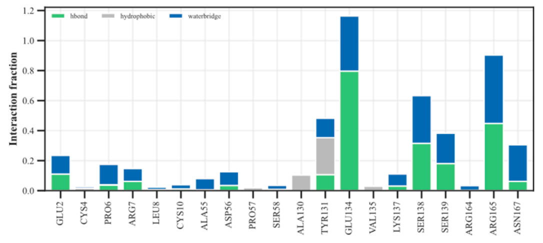	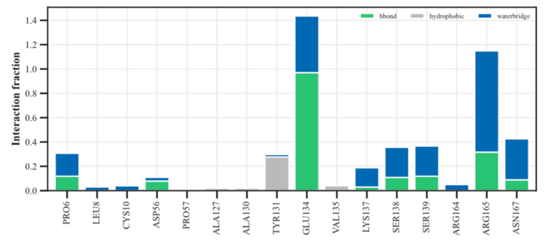

**Ginsenoside Rb1 (Site 2)**	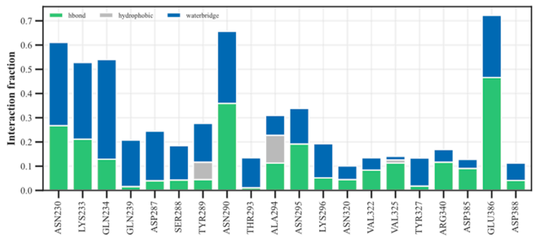	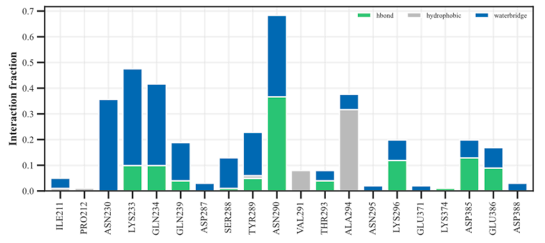
**Ginsenoside Rb1 (Site 3)**	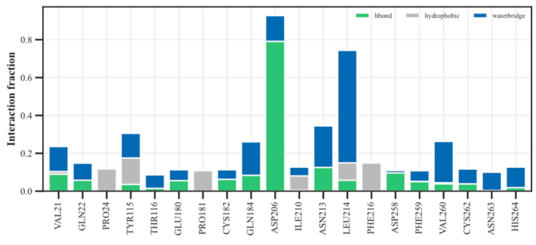	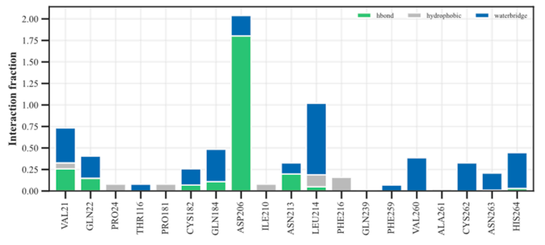
**Ginsenoside Re (Site 1)**	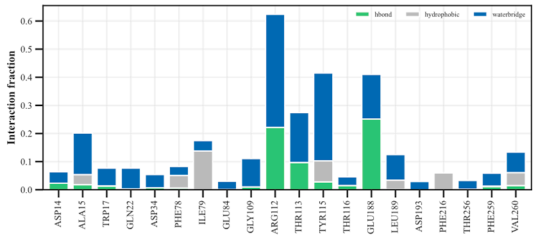	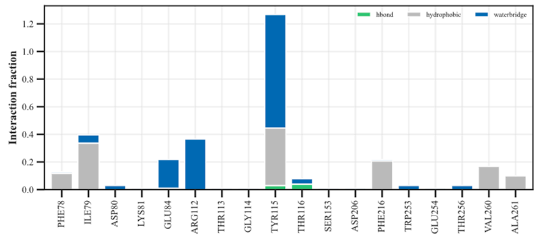
**Ginsenoside Re (Site 2)**	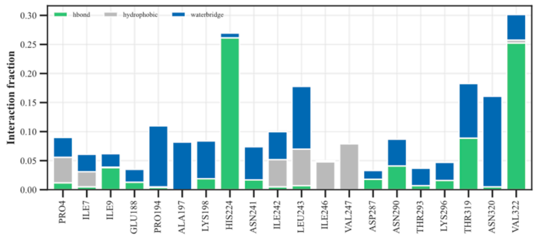	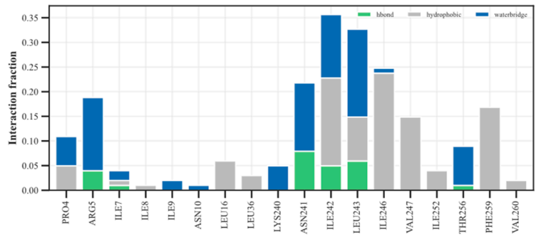
**Ginsenoside Re (Site 3)**	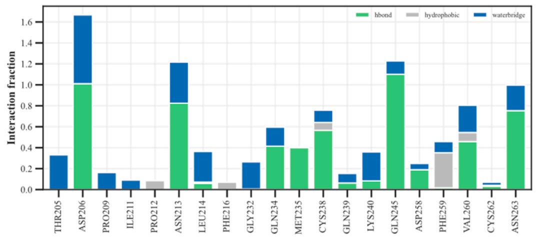	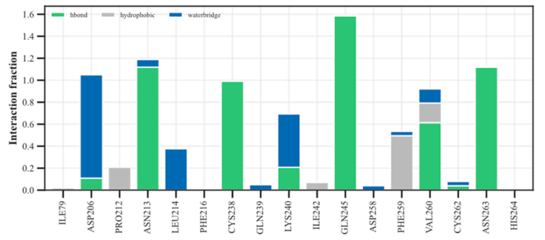
**Ginsenoside Rd (Site 1)**	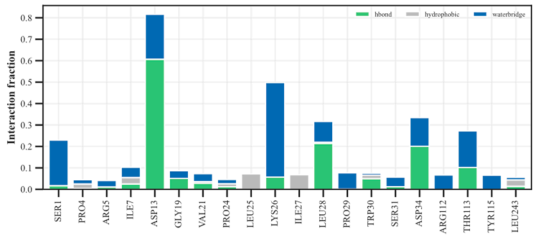	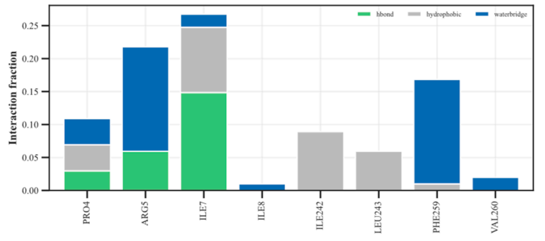
**Ginsenoside Rd (Site 2)**	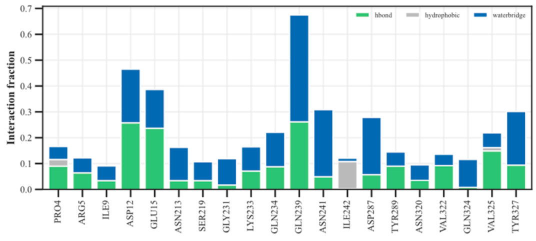	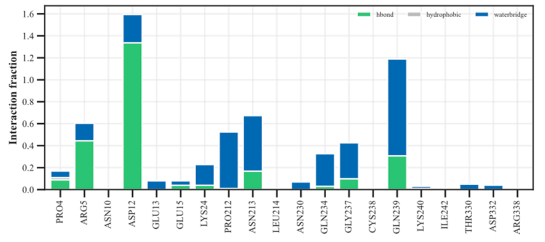

**Ginsenoside Rd (Site 3)**	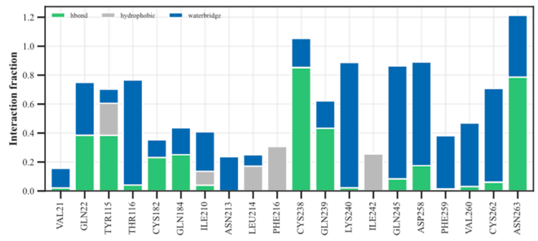	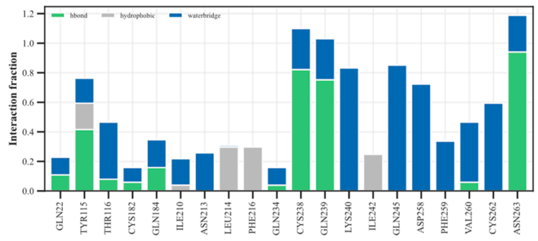
**Ginsenoside Rg1 (Site 1)**	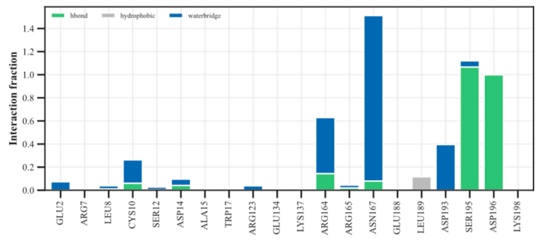	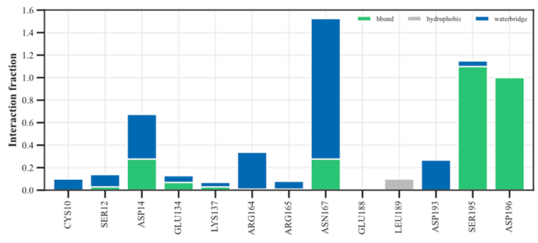
**Ginsenoside Rg1 (Site 2)**	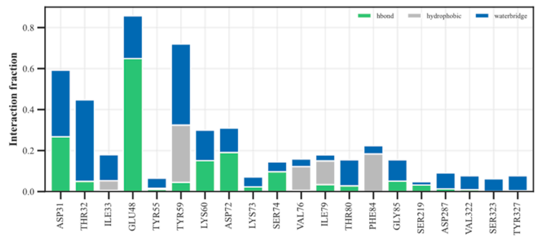	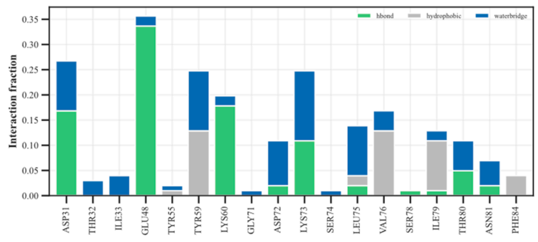
**Ginsenoside Rg1 (Site 3)**	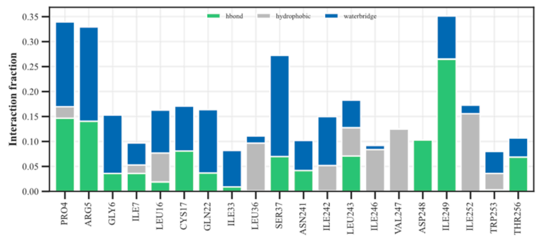	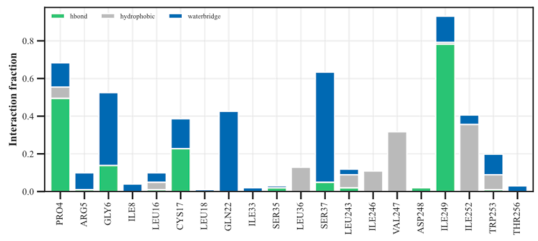
**Osladin (Site 1)**	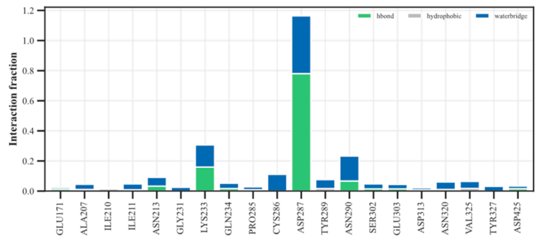	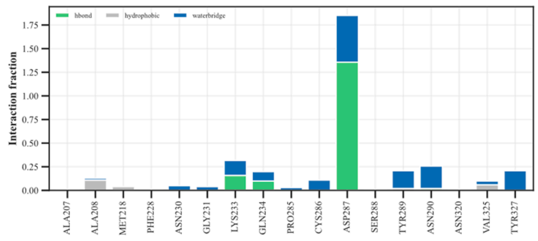
**Osladin (Site 2)**	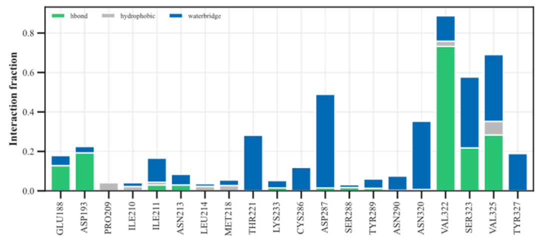	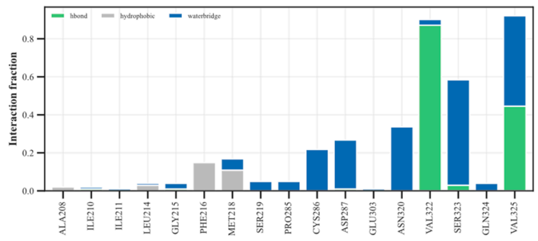
**Osladin (Site 3)**	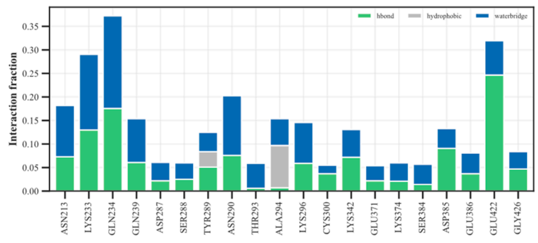	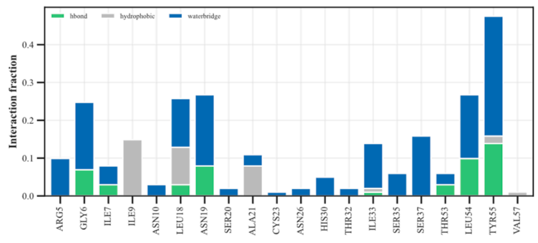

**Herniariasaponin 2 (Site 1)**	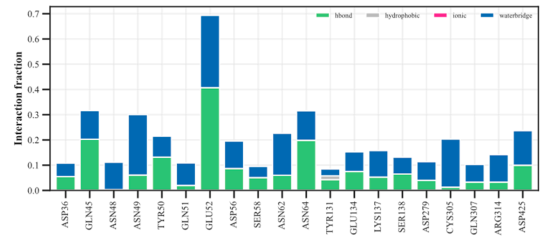	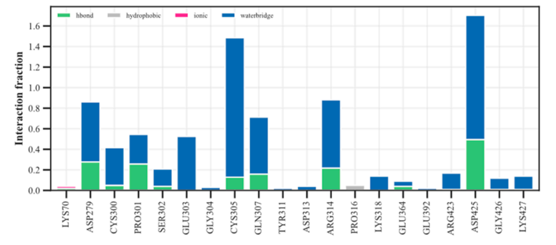
**Herniariasaponin 2 (Site 2)**	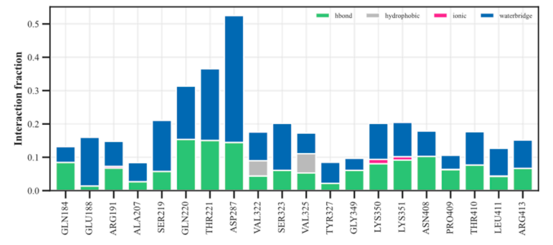	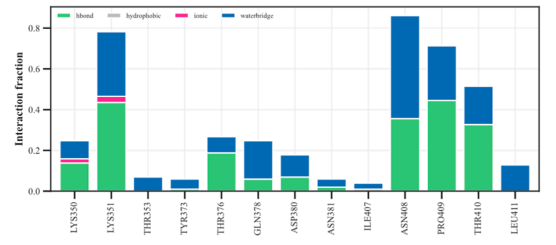
**Herniariasaponin 2 (Site 3)**	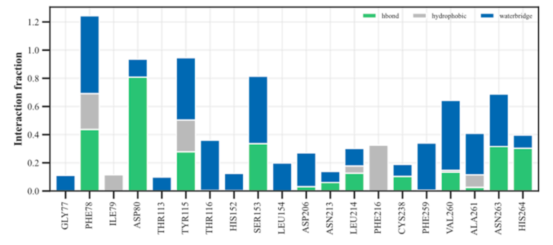	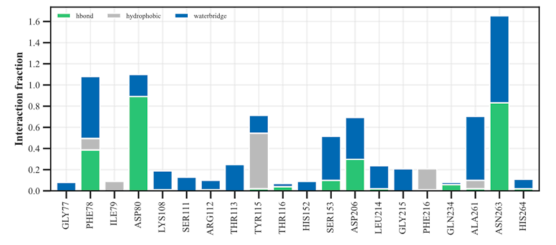
**Herniariasaponin 4 (Site 1)**	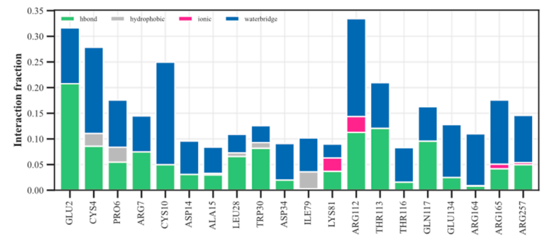	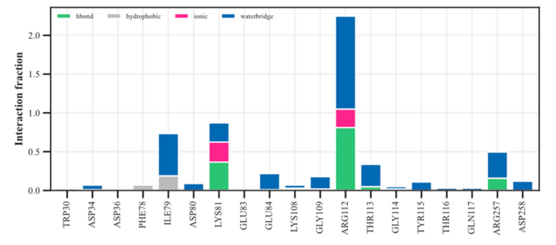
**Herniariasaponin 4 (Site 2)**	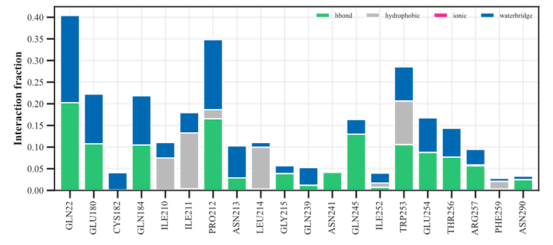	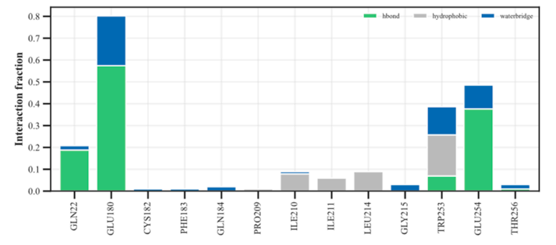
**Herniariasaponin 4 (Site 3)**	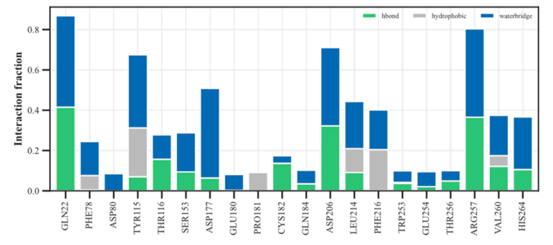	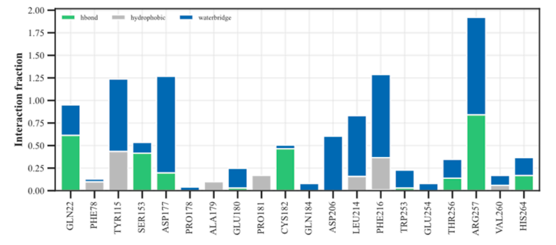

Figure S4. Ligand Root Mean Square Deviation (RMSD) values throughout a 500 ns molecular dynamics simulation for saponin-lipase-colipase complexes.

Ligand	RMSD (site 1)	RMSD (site 2)	RMSD (site 3)
**Cholate**	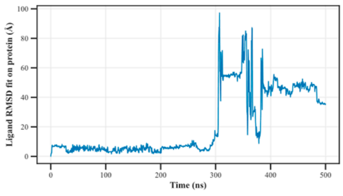	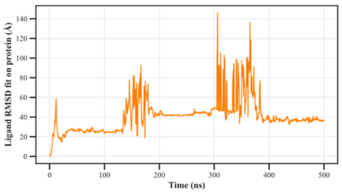	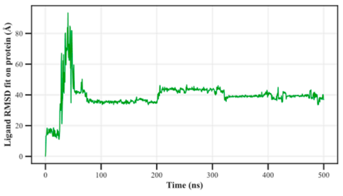
**Escin Ia**	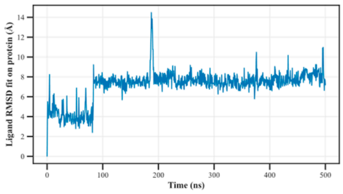	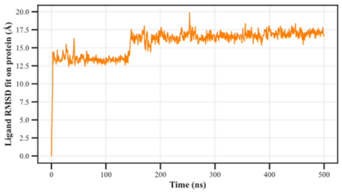	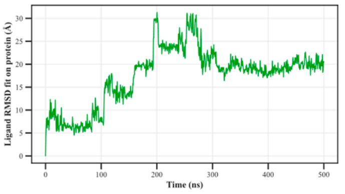
**Hederacoside**	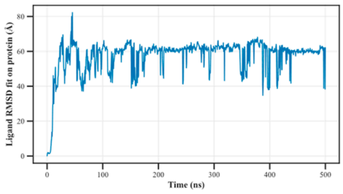	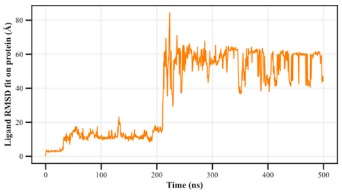	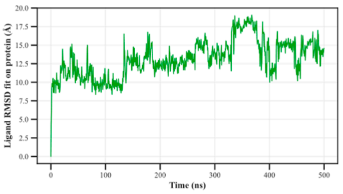
**Ginsenoside Rb1**	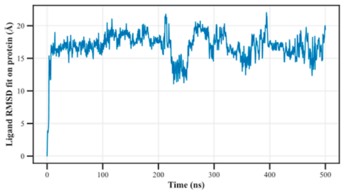	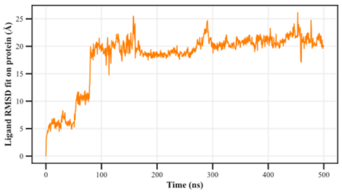	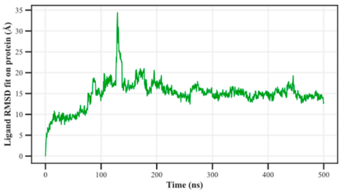
**Ginsenoside Re**	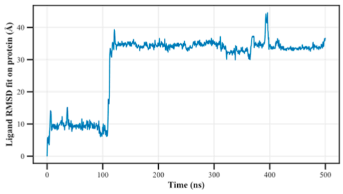	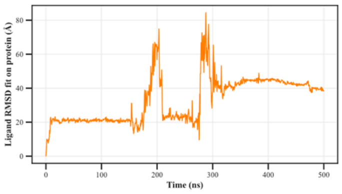	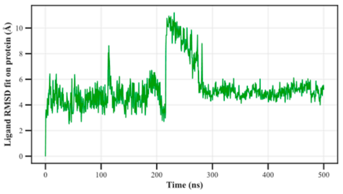
**Ginsenoside Rd**	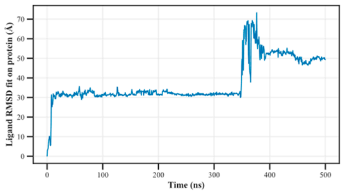	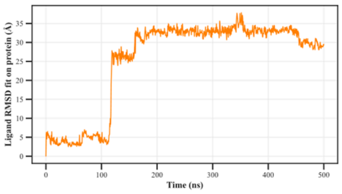	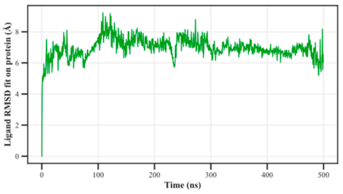
**Ginsenoside Rg1**	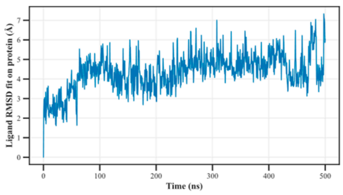	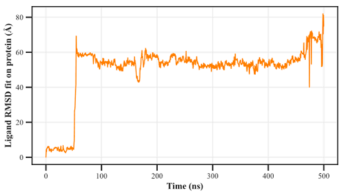	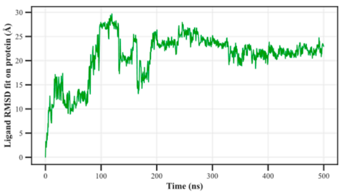
**Osladin**	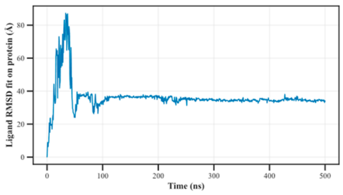	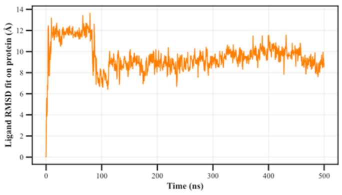	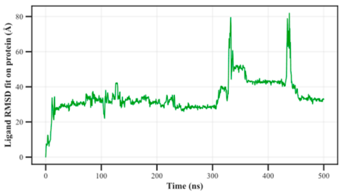

**Herniariasaponin 2**	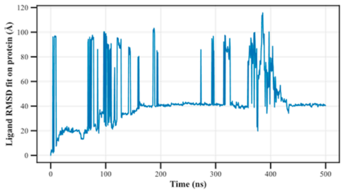	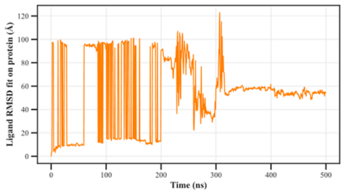	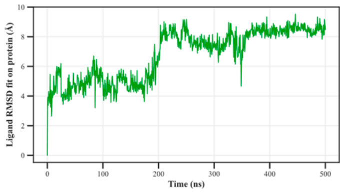
**Herniariasaponin 4**	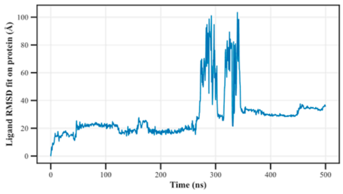	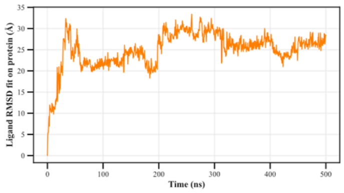	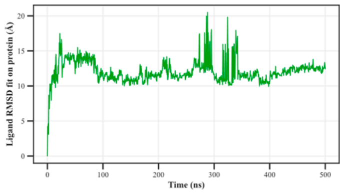

Figure S5. Ligand Root Mean Square Fluctuation (RMSF) values throughout a 500 ns molecular dynamics simulation for saponin-lipase-colipase complexes.

Ligand	RMSF (site 1)	RMSF (site 2)	RMSF (site 3)
**Cholate**	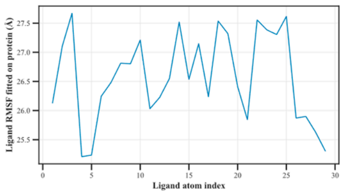	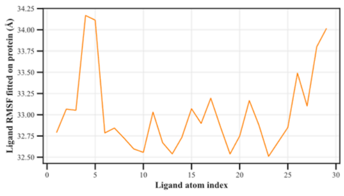	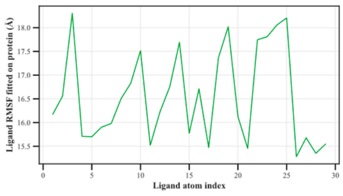
**Escin Ia**	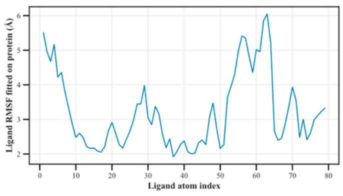	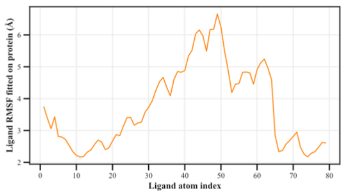	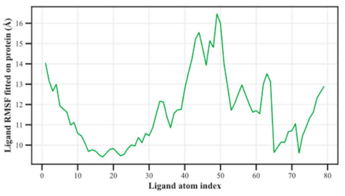
**Hederacoside**	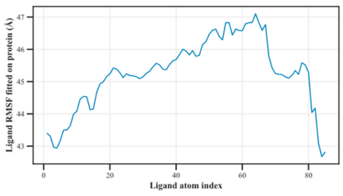	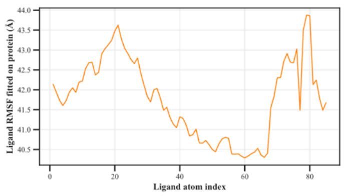	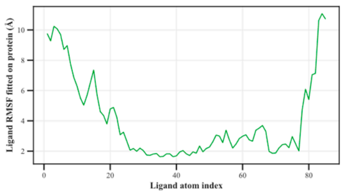
**Ginsenoside Rb1**	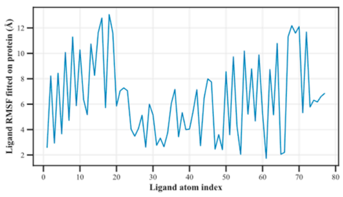	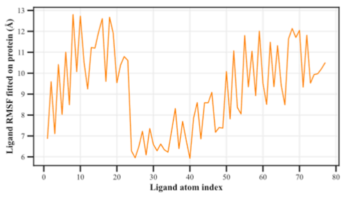	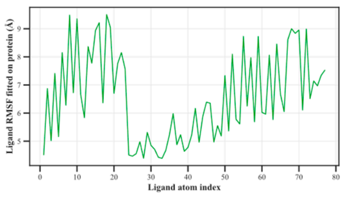
**Ginsenoside Re**	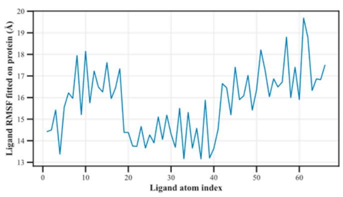	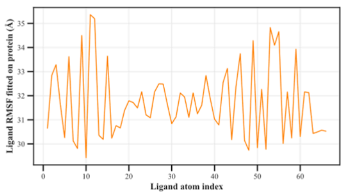	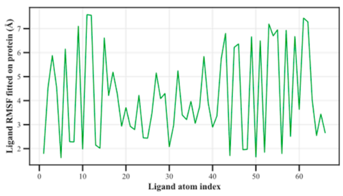

**Ginsenoside Rd**	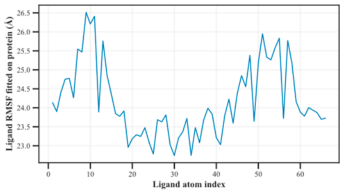	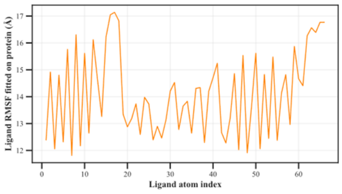	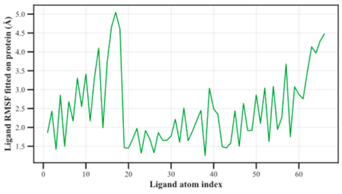
**Ginsenoside Rg1**	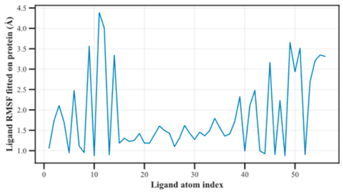	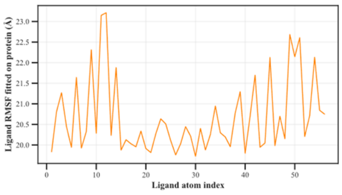	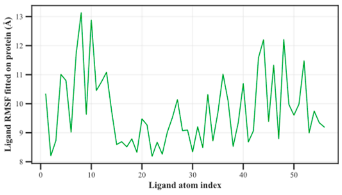
**Osladin**	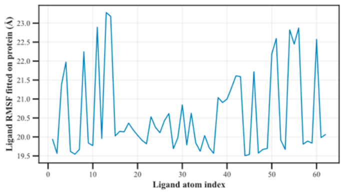	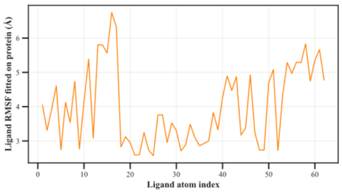	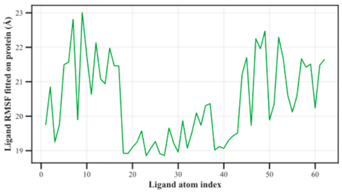
**Herniariasaponin 2**	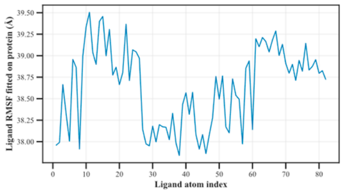	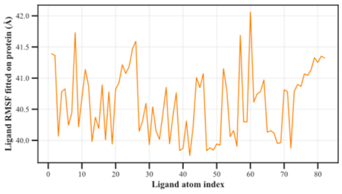	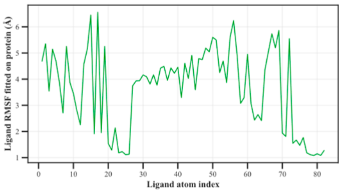
**Herniariasaponin 4**	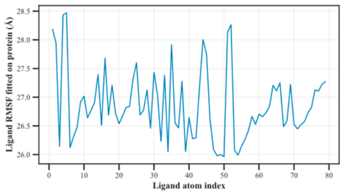	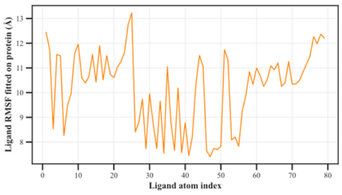	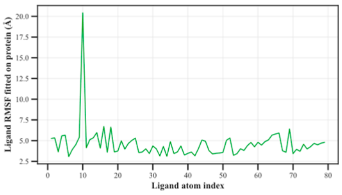
